# iPCD: A Comprehensive Data Resource of Regulatory Proteins in Programmed Cell Death

**DOI:** 10.3390/cells11132018

**Published:** 2022-06-24

**Authors:** Dachao Tang, Cheng Han, Shaofeng Lin, Xiaodan Tan, Weizhi Zhang, Di Peng, Chenwei Wang, Yu Xue

**Affiliations:** Key Laboratory of Molecular Biophysics of Ministry of Education, Hubei Bioinformatics and Molecular Imaging Key Laboratory, Center for Artificial Intelligence Biology, College of Life Science and Technology, Huazhong University of Science and Technology, Wuhan 430074, China; tangdc@hust.edu.cn (D.T.); hancheng@hust.edu.cn (C.H.); linshaofeng@hust.edu.cn (S.L.); tanxiaodan@hust.edu.cn (X.T.); u201512163@hust.edu.cn (W.Z.); pengdi@hust.edu.cn (D.P.)

**Keywords:** programmed cell death, PCD protein, apoptosis, ferroptosis, GPX4

## Abstract

Programmed cell death (PCD) is an essential biological process involved in many human pathologies. According to the continuous discovery of new PCD forms, a large number of proteins have been found to regulate PCD. Notably, post-translational modifications play critical roles in PCD process and the rapid advances in proteomics have facilitated the discovery of new PCD proteins. However, an integrative resource has yet to be established for maintaining these regulatory proteins. Here, we briefly summarize the mainstream PCD forms, as well as the current progress in the development of public databases to collect, curate and annotate PCD proteins. Further, we developed a comprehensive database, with integrated annotations for programmed cell death (iPCD), which contained 1,091,014 regulatory proteins involved in 30 PCD forms across 562 eukaryotic species. From the scientific literature, we manually collected 6493 experimentally identified PCD proteins, and an orthologous search was then conducted to computationally identify more potential PCD proteins. Additionally, we provided an in-depth annotation of PCD proteins in eight model organisms, by integrating the knowledge from 102 additional resources that covered 16 aspects, including post-translational modification, protein expression/proteomics, genetic variation and mutation, functional annotation, structural annotation, physicochemical property, functional domain, disease-associated information, protein–protein interaction, drug–target relation, orthologous information, biological pathway, transcriptional regulator, mRNA expression, subcellular localization and DNA and RNA element. With a data volume of 125 GB, we anticipate that iPCD can serve as a highly useful resource for further analysis of PCD in eukaryotes.

## 1. Introduction

Programmed cell death (PCD) is an evolutionarily conserved process in multicellular organisms that precisely controls the suicide of specific cells under developmental or environmental stimuli. Dysregulation of PCD is closely associated with human diseases, such as cancer, neurodegenerative diseases and metabolic disorders [1,2,3,4,5,6]. Various PCD pathways play a critical role in the fight of the host to combat cancer and pathogen infections by activating immune system responses. A large number of important proteins have been identified to regulate PCD and mediate the communications and crosstalk among different PCD forms. In this regard, the identification of regulatory proteins involved in PCD is the foundation for understanding the molecular mechanisms and dynamics of PCD, thus, providing potential targets for further drug design [7,8,9].

According to in-depth exploration of PCD regulatory mechanisms, an increasing number of new PCD forms have been discovered. Apoptosis, the first identified PCD form in metazoans and plants, was reported to physiologically disassemble cells into apoptotic bodies for elimination in a caspase-dependent manner [10]. From human follicular lymphoma, BCL-2 was identified as the first apoptotic regulator and was also found to be involved in the regulation of other PCD forms, such as necroptosis, autophagy, mitotic catastrophe and pyroptosis [1,11,12]. In contrast with conventional thinking as an accidental way for cells to die, necrosis has emerged as a regulated cell death form, necroptosis, which is activated by RIPK3-mediated MLKL oligomerization [13]. Although autophagy has been generally regarded as a regulatory mechanism for cell survival, massive autophagy can trigger a distinct PCD form, autophagic cell death [14]. Additionally, pyroptosis has been newly identified as a novel PCD form, which is regulated by caspase-1-mediated cleavage and activation of gasdermin D (GSDMD) [15]. More recently, ferroptosis has been reported as a novel iron-dependent PCD form that plays a vital role in many diseases, and ferroptosis is induced by excessive lipid peroxidation [16,17]. In addition, other PCD forms have been discovered, such as anoikis [18], hypersensitive response (HR) [19] and mitotic catastrophe [20].

Given the importance of different PCD forms in health and disease, tremendous efforts have been made to identify and integrate crucial PCD protein regulators. In the past two decades, multiple databases have been constructed for the collection of PCD regulators, including DeathBase [21], yApoptosis [22], Cell Death Proteomics database (CDP) [23], Autophagy Database [24], Human Autophagy Database (HADb) [25], Autophagy Regulatory Network (ARN) [26], Human Autophagy Modulator Database (HAMdb) [27], Autophagy and Tumor Database (ATdb) [28], FerrDb [29], MCDB [30], miRDeathDB [31] and ncRDeathDB [32] (Table S1). These data resources provide valuable clues for further deciphering PCD systems. In contrast to the growing number of PCD forms, existing databases only contain limited regulatory proteins from a handful of PCDs. In addition, comprehensive annotations for these regulatory proteins are still lacking. Therefore, the development of a comprehensive database for the integration of known PCD proteins as well as corresponding information is urgently needed.

In this study, we first summarized the mainstream data resources for PCD that have been maintained and devoted for the academic community. Through literature biocuration and the incorporation of public resources, we developed a new database, integrated annotations for programmed cell death (iPCD, http://ipcd.biocuckoo.cn/, accessed on 25 June 2021), which contains 1,091,014 known and computationally identified protein regulators from 30 major forms of PCD, including apoptosis [10], necroptosis/necrosis [33], autophagic cell death/autophagy [34], ferroptosis [16], anoikis [35], pyroptosis [36], oncosis [37], paraptosis [38], parthanatos [39], cytotoxic granule-mediated cell death [40], oxeiptosis [41], pyronecrosis [42], alkaliptosis [43], autosis [44], lysosomal cell death/autolysis [45], cornification [46], entosis [47], HR [19], mitoptosis [48], excitotoxicity [49], NETosis [50], phenoptosis [51], heterokaryon incompatibility (HI) [52], mitotic catastrophe [20], sarmoptosis [53], Wallerian degeneration [54], oxiapoptophagy [55], immunogenic cell death [56], phagoptosis [45] and PANoptosis [57], in 562 eukaryotic species. To extend the application of our data, rich annotations were subsequently provided for PCD regulators of eight model species from 16 aspects, which contained 102 additional public resources. Based on systematic integration, 1,091,014 PCD proteins from 30 PCD forms were collected in iPCD, and at least a 600-fold increase in data size (125 GB) compared to other PCD databases. Additionally, iPCD will be continuously maintained and updated. We anticipate that iPCD can serve as a highly helpful resource for the analysis of PCD in eukaryotes and further contribute to the treatment of diseases.

## 2. Materials and Methods

### 2.1. Data Collection and Curation

**Figure 1:**
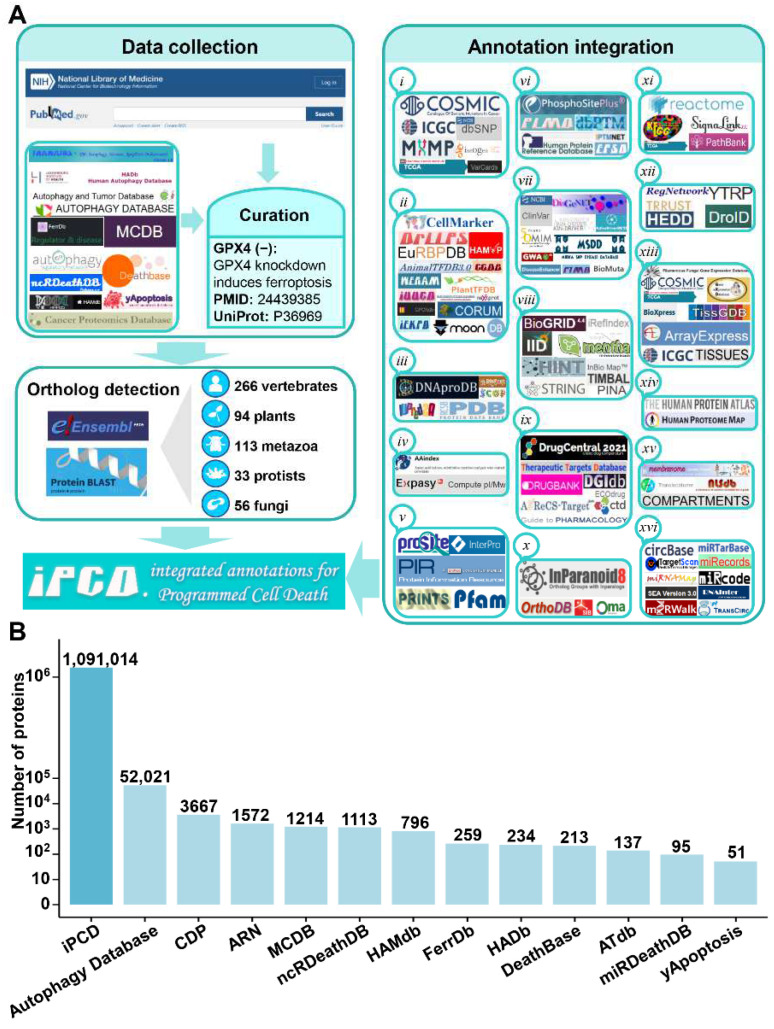
The procedure for the construction of iPCD and the comparison between iPCD and 12 additional databases. (**A**) The workflow for the construction of the iPCD database. First, we collected 30 experimentally identified forms of PCD proteins from PubMed and annotated them ‘+’ or ‘−’ as positive or negative PCD regulator. Then, 11 additional PCD databases were merged and carefully re-curated for each entry, and further conducted an orthologous search to computationally identify new PCD regulators in 562 eukaryotes. Next, the PCD regulators were annotated with 102 additional public resources in 8 model organisms that covered 16 aspects. (**B**) The numbers of PCD proteins included in iPCD and 13 additional databases.

In this study, we first collected experimentally identified proteins from PubMed by using multiple keywords, such as ‘apoptosis’, ‘apoptotic’, ‘anoikis’, ‘cytotoxic granule-mediated cell death’, ‘oxeiptosis’, ‘necroptosis’, ‘necrosis’, ‘necrotic’, ‘pyroptosis’, ‘oncosis’, ‘oncotic’, ‘pyronecrosis’, ‘alkaliptosis’, ‘autophagic’, ‘autophagy’, ‘autosis’, ‘lysosomal cell death’, ‘autolysis’, ‘cornification’, ‘entosis’, ‘entotic’, ‘ferroptosis’, ‘hypersensitive response’, ‘mitoptosis’, ‘paraptosis’, ‘excitotoxicity’, ‘NETosis’, ‘netotic’, ‘parthanatos’, ‘phenoptosis’, ‘heterokaryon incompatibility’, ‘mitotic catastrophe’, ‘Wallerian degeneration’, ‘oxiapoptophagy’, ‘immunogenic cell death’, ‘phagoptosis’, ‘sarmoptosis’ and ‘PANoptosis’. In addition, we also integrated downloadable data resources from 11 PCD databases, including DeathBase [21], yApoptosis [22], CDP [23], Autophagy Database [24], HADb [25], ARN [26], HAMdb [27], ATdb [28], FerrDb [29], MCDB [30] and ncRDeathDB [32] (Figure 1A, Supplementary Methods). In order to ensure the quality of data, we carefully read the abstracts and the full text of papers. Specifically, the merged data from 11 databases were rechecked by searching PubMed, and only data supported by the literature were retained. Compared to 11 other PCD databases, the number and distribution of newly collected PCD regulators identified from experiments are shown in Table S2. The experimental evidence for each protein was preserved, and all these proteins were denoted as ‘Reviewed’ in iPCD. For each protein entry, the positive or negative PCD regulations were tagged as ‘+’ or ‘−’, respectively. For example, the knockdown of human protein GPX4 can induce ferroptosis [58]. Therefore, GPX4 protein is tagged with ‘−’ as a negative regulator of ferroptosis (Figure 1A).

### 2.2. Genome-Wide Identification

The potential orthologs of known PCD-associated proteins were detected and identified in other species (Figure 1A). The complete proteome sets of 562 eukaryotes were downloaded, including 266 vertebrates from Ensembl (release version 103, http://www.ensembl.org/, accessed on 13 April 2021), 94 plants from EnsemblPlants (release version 50, http://plants.ensembl.org/, accessed on 13 April 2021), 113 metazoa from EnsemblMetazoa (release version 50, http://metazoa.ensembl.org/, accessed on 13 April 2021), 33 protists from EnsemblProtists (release version 50, http://protists.ensembl.org/, accessed on 13 April 2021) and 56 fungi from EnsemblFungi (release version 50, http://fungi.ensembl.org/, accessed on 13 April 2021). To eliminate the redundancy, the Ensembl Gene ID was used as the primary accession. Since one gene could derive multiple alternatively splicing isoforms, only the longest protein and its corresponding genomic sequence was reserved to avoid redundancy. Furthermore, the method of reciprocal best hits (RBHs) was adopted to find orthologous pairs in two organisms, and the blastall program in the BLAST package was chosen to detect potential orthologs [59], with a stringent threshold of *E*-value ≤ 10^−8^. The default amino acid substitution matrix, BLOSUM62, was adopted, and the low-complexity regions were masked during the BLAST search. A pair of potential orthologs was identified and retained if the two proteins in two different proteomes reciprocally found each other as the first best hit. Figure 1B clearly shows the difference in the numbers of PCD proteins for iPCD and 12 additional databases. PCD forms and species were also counted and displayed (Figure S1).

### 2.3. Annotation of Regulatory Proteins

The PCD regulators were collected and annotated. First, 1,091,014 PCD regulators were annotated with basic information, including ‘iPCD ID’, ‘status’, ‘Ensembl Gene ID’, ‘Ensembl Protein ID’, ‘UniProt Accession’, ‘PDB structure’, ‘Function’, ‘Protein Sequence’, ‘Nucleotide Sequence’, ‘Key word’ and ‘Gene Ontology’. For each species, all regulatory proteins were alphabetically ordered based on their gene names. Then, each protein was automatically assigned a unique iPCD ID for convenient organization in the database. For example, the GPX4 protein in *Homo sapiens* was assigned with an ID of ‘iPCD-Hsa-1522’. Subsequently, 102 additional public resources were integrated to annotate 17,768 regulators from 8 model organisms, including *Homo sapiens*, *Mus musculus*, *Rattus norvegicus*, *Drosophila melanogaster*, *Caenorhabditis elegans*, *Saccharomyces cerevisiae*, *Arabidopsis thaliana* and *Danio rerio*. These public resources covered 16 distinct aspects, which contained (*i*) genetic variation and mutation, (*ii*) functional annotation, (*iii*) structural annotation, (*iv*) physicochemical property, (*v*) functional domain, (*vi*) post-translational modification (PTM), (*vii*) disease-associated information, (*viii*) protein–protein interaction (PPI), (*ix*) drug-target relation, (*x*) orthologous information, (*xi*) biological pathway, (*xii*) transcriptional regulator, (*xiii*) mRNA expression, (*xiv*) protein expression/proteomics, (*xv*) subcellular localization and (*xvi*) DNA and RNA element. The detailed processing of 102 public resources is provided in Supplementary Methods. A brief flowchart of the study is shown in Figure 1A.

## 3. Results

### 3.1. A Brief Summarization of Different PCD Forms

The concept of PCD has been established for more than 50 years [60,61,62]. Currently, PCD is one of most extensively studied processes in cell biology. At least 30 PCD forms have been discovered, and detailed information of each PCD form is provided in Table S3. For convenient organization in our database, we classified known PCD forms into six categories, including apoptotic cell death (ACD), necrotic cell death (NCD), autophagy-dependent cell death (ADCD), other lysosomal cell death (OLCD), mitochondria-associated cell death (MCD) and unclassified cell death (UCD), mainly based on similar cell death morphology, or the key organelle required for orchestrating PCD. 

*(i)* ACD comprises the canonical apoptosis and other apoptosis-like PCD forms. Apoptosis is the first identified PCD reported by John Kerr and his coworkers in 1972 [10]. Apoptosis has unique morphological characteristics and molecular mechanisms that are mediated by a series of caspases, and BCL-2 family proteins act as negative regulators to inhibit apoptosis. In 1994, Steven M. Frisch and Hunter Francis introduced ‘anoikis’ to describe a new PCD form, morphologically similar to apoptosis [35]. Anoikis is triggered by the disruption of the epithelial cell–matrix interactions, and BCL-2 family proteins also confer resistance to anoikis. Meanwhile, granules secreted by cytotoxic T lymphocytes can induce a novel form of PCD, called ‘cytotoxic granule-mediated cell death’ or ‘cytotoxic granule-mediated apoptosis’, which is molecularly, structurally and functionally different from but morphologically similar to canonical apoptosis. In contrast to the canonical apoptosis regulated by caspases and BCL-2 family proteins, perforin and granzymes are the key molecules in the signaling pathway of cytotoxic granule-mediated cell death [40,63,64]. In 2018, Cathleen Holze identified a novel reactive oxygen species (ROS)-induced PCD, oxeiptosis, by ozone exposure in a mouse model. Oxeiptosis has an apoptosis-like morphology and a caspase-independent signaling pathway. Oxeiptosis can be triggered by the activation of the KEAP1-PGAM5-AIFM1 molecular pathway [41].

*(ii)* NCD comprises necrosis/necroptosis and other regulated necrosis-like PCD forms. Necrosis was usually regarded as accidental or unregulated cell death for a long time before the term ‘necroptosis’ was defined by Yuan and her colleagues, which indicates that necrosis is a form of PCD [33,65]. Necroptosis is triggered by the activation and interaction of RIPK1, RIPK3 and MLKL [66]. In 2001, the term ‘pyroptosis’ was proposed by Brad T. Cookson and Molly A. Brennan to describe a process of proinflammatory PCD [36]. Pyroptosis is morphologically similar to necroptosis [67] and can be driven by the activation of Caspase-1 and mediated by nonselective GSDMD pores [68,69]. The importance of pyroptosis emerged when its important role in the regulation of inflammation and immunity was revealed. Meanwhile, oncosis is a form of NCD induced by energy depletion and cell swelling. Oncosis was originally considered an unregulated form of necrosis until a host of reports demonstrated the importance of the PORIMIN receptor [37] and uncoupling protein 2 (UCP-2) [70] in the regulation of oncosis. Additionally, in contrast to pathogen-induced programmed necrosis in plants, the crucial role of CIAS1/cryopyrin/NLRP3 and ASC in microbial pathogen-induced NCD in animals was illustrated by Willingham, and the term ‘pyronecrosis’ was coined in 2007 [42]. More recently, Song et al. described ‘alkaliptosis’ as a pH-dependent form of NCD that occurred in pancreatic ductal adenocarcinoma cancer cells in 2018. JTC801 induces alkaliptosis by intracellular alkalinization, and the molecular machinery of alkaliptosis is dependent on the downregulation of CA9 by the activation of NF-κB [43].

*(iii)* ADCD is a lysosome-dependent PCD form. Macroautophagy (hereafter called ‘autophagy’) is a highly conserved process to degrade cellular contents in eukaryotes, while excessive autophagy will also give rise to autophagic cell death [34]. To date, 43 autophagy-related genes (*ATGs*) have been identified [71], which may also play key roles in autophagic cell death. In 2013, Liu et al. identified a novel form of PCD, autosis, as a subtype of autophagic cell death that can be triggered by Tat-Beclin 1, starvation or hypoxia-ischemia [44]. Autosis is regulated by Na^+^, K^+^-ATPase, which maintains ion homeostasis as a plasma pump and is linked to ER stress.

*(iv)* OLCD comprises other forms of lysosome-dependent PCD beyond ADCD. These PCD forms are molecularly and morphologically different. However, lysosome is the key organelle required for orchestrating these PCD processes. The first form of OLCD, lysosomal cell death, was first reported by Franko and coworkers in 2000 [72]. Further, hydrolases, such as cathepsins released by lysosomal membrane permeabilization, contribute to lysosomal cell death, also known as autolysis, and have been defined as a form of OLCD [45]. Moreover, the terminal differentiation of keratinocytes in the epidermis, cornification, was considered as a specific PCD form [46]. In 2005, Eleonora Candi described cornification as a model of cell death and emphasized the significant roles of transglutaminases in this process. Of note, lysosomal proteases play critical roles in cornification [73]. Next, the cell death of human tumors induced by a cell-in-cell invasion structure or cell cannibalism was first observed by Michael Overholtzer in 2007, and this process was assigned the name ‘entosis’ with distinguishable cytological features [47]. Entotic cell death is closely related to the lysosomal degradation pathway. Later, erastin treatment triggered individual regulated cell death with iron accumulation and lipid peroxidation. Thus, ‘ferroptosis’ was proposed by Dixon and coworkers to depict this death phenotype in 2012 [16]. Ferroptosis is a novel PCD with unique morphological, genetic and biochemical characteristics that are distinct from apoptotic, necrotic and autophagic cell death pathways, and the inhibition of GPX4 can effectively induce ferroptosis [69]. Lysosome is crucial for regulating iron metabolism in ferroptosis [74].

*(v)* MCD comprises molecularly and morphologically different PCD forms associated with mitochondria. For example, the infection of microbial pathogens gives rise to the response of host plant cells, often accompanied with cell death in and around the attempted pathogen invasion site, and this process is termed ‘hypersensitive response’. Death signaling triggers HR through a mitochondrial action [19,75]. Direct or indirect activation of the core molecular NLRs can cause HR cell death. In 1999, Vladimir P. Skulachev first introduced the term ‘mitoptosis’ as a typical form of MCD [48]. Later, Sperandio et al. proposed ‘paraptosis’ as a non-apoptotic form of PCD in 2000 [38]. The activity of paraptosis induced by IGFIR is dependent on the mediation of mitogen-activated protein kinases (MAPKs), and it can be inhibited by overexpression of AIP1/Alix [76]. Further studies demonstrated that the mitochondrial dysfunction contributes to the induction of paraptosis [77]. In addition, a form of PCD in neurons derived from high levels of glutamate and Ca^2+^ overload is known as ‘excitotoxicity’. The occurrence of excitotoxicity depends on the activation of NMDA receptors, when the mitochondrial function is disrupted by excess calcium uptake [49]. In 2004, ‘NETosis’ was first discovered by Volker Brinkmann. Neutrophil extracellular traps (NETs) are generated by activating neutrophils to digest virulence factors and kill pathogens in response to infection or injury [50]. PCD triggered by the formation and release of NETs is named ‘netotic cell death’. ROS produced in mitochondria promote the induction of NETosis caused by various stimuli [78]. Moreover, Parthanatos is a PARP1-dependent PCD caused by the release of AIF from mitochondria into the nucleus [39]. Parthanatos is associated with necrosis and apoptosis, but it has a distinct molecular mechanism.

*(vi)* UCD comprises other molecularly and morphologically different PCD forms that could not be classified in any of the above five categories. The word ‘phenoptosis’ was described as a PCD form by Vladimir P. Skulachev in 1997 [51]. Meanwhile, the fusion of incompatible genotypes leads heterokaryotic cells to be quickly compartmentalized and subsequently undergo PCD, which is known as ‘heterokaryon incompatibility’ [52,79,80]. In addition, a special case of cell death derived from mitotic arrest is called the ‘mitotic catastrophe’ [20]. The combination of specific types of cell damage and deficient cell-cycle checkpoints can lead to mitotic catastrophe, such as DNA damage and the genetic suppression of G2 checkpoint genes. The Cdk1/cyclin B1 complex is reported to be essential for mitotic catastrophe [81]. In addition, Wallerian degeneration is an emerging PCD. Nerve fibers are broken in the process of Wallerian degeneration, with subsequent neuron axon degeneration. Wallerian degeneration requires NMNATs, SARM1 and PHR1, which are the core components of the axon death pathway [54,82]. Apart from this, some oxysterols can trigger a distinctive form of cell death that combines oxidative stress, apoptosis and autophagy, coined ‘oxiapoptophagy’, which was introduced in 2003 [55]. In 2005, a caspase-dependent form of cell death was found to stimulate an immune response against dead-cell antigens in tumor cells, which was termed ‘immunogenic cell death’ [56,83]. Phagoptosis is a recognized form of PCD that can execute the death of viable cells by phagocytosis [84] and consists of three main steps: recognition, engulfment and digestion. CD47 plays a crucial role in the signaling pathway of phagoptosis [45]. Sarmoptosis is a novel sarm1-dependent form of PCD in sensory neurons associated with mitochondrial dysfunction [53]. More recently, PANoptosis has become known as an inflammatory form of cell death that highlights the crosstalk and coordination of pyroptosis, apoptosis and necroptosis [57]. The PANoptosome is the critical component that drives PANoptosis.

### 3.2. A Summary of Mainstream Databases for PCD

In the past few decades, a large number of proteins and genes have been identified to participate in or regulate the PCD process in numerous experimental studies. However, it is still a great challenge to gather, curate and annotate these cumulative data for further experimental and computational applications. The apoptosis database, constructed by Doctor and coworkers in 2003, is the first PCD database and aimed to gather apoptosis-associated proteins with their distinctive structural domains [85]. In 2010, Díez et al. developed a valuable database, DeathBase, which focuses on apoptosis and contains 213 apoptosis proteins from five model species [21]. Later, Wanichthanarak et al. released a high-quality yeast apoptosis database, named yApoptosis, which collects 51 apoptosis-associated proteins and depicts gene interaction networks [22]. With advances in high-throughput mass spectrometry (HTP-MS), quantitative proteomics is now becoming an important tool to analyze differential protein expression under various PCD conditions [86]. In 2012, Magnus et al. constructed the well-organized database APdb, which integrated cell apoptosis proteomics data [87]. Additionally, the comprehensive protein proteomics database CDP was created, which consists of eight PCD forms and 3667 proteins’ potential interaction with PCD [23].

In addition, Homma et al. built the first autophagy database in 2010, which maintains a total of 2163 autophagy proteins or homologs in 41 eukaryotes. Later, the Autophagy database was updated and now contains 52,021 protein entries [24]. In parallel, Moussay et al. developed a HADb database by collecting 222 human genes directly or indirectly related to autophagy in 2011 [25]. With the accumulation of autophagy protein regulators, studies on the interaction mechanism between autophagy regulators have emerged. In 2015, Türei et al. set up a high-quality database, ARN, which contains 1485 proteins and 4013 interactions [26]. Recently, they updated the ARN database to version 1.0.6, which included 1572 autophagy regulators with 4523 interactions. Moreover, due to the strong link between PCD and disease, Wang et al. developed a comprehensive database, HAMdb, covering 796 proteins, 841 chemicals and 132 microRNAs, to provide the function, pathway and disease information of autophagy modulators [27]. In addition, the ATdb database integrated 25 types of tumors and 137 autophagy genes for the investigation of the association between autophagy and tumors [28].

More recently, Zhou et al. developed the first ferroptosis database, FerrDb, which contains 253 ferroptosis regulators, 111 makers and 95 ferroptosis-disease associations [29]. In 2021, Zhang et al. reported the first mitotic catastrophe database, MCDB, which contains 1214 proteins or genes and 5014 compounds [30]. Since the differential expression of PCD was observed in different systems, the integration of transcriptional information of PCD is also needed. In 2012, Xu et al. built an excellent database, miRDeathDB, to investigate the crucial role of microRNAs in the processes of apoptosis, autophagy and programmed necrosis, which consists of 86 miRNAs and 95 genes in five species [31]. Later, they developed the ncRDeathDB to archive ncRNA-regulated cell death systems in 2015, with more than 4600 ncRNA-mediated PCD-related entries in 12 species, collected and organized [32]. Based on existing known PCD data, some useful computational tools have been released for the efficient identification and calculation of cell death. In 2015, Daniel et al. developed a novel ImageJ macro to quantitate retinal cell death based on the TUNEL assay [88]. Recently, we constructed DeepPhagy to quantitatively measure autophagy activity in *S. cerevisiae* based on a deep learning framework [89]. Altogether, these databases or tools provide valuable information to further promote the investigation of PCD in eukaryotes. A brief summary of the 12 PCD databases is shown in Table S1.

### 3.3. The Multi-Layer Annotation of PCD Regulatory Proteins

In this study, we developed iPCD as a protein-centered database. The iPCD ID was used to organize the database, which was automatically generated as the primary accession for each PCD regulator. For example, the *Homo sapiens* protein GPX4 was assigned to ‘iPCD-Hsa-1522’. First, the basic annotation information of each protein was obtained directly from UniProt [90], including protein name, UniProt accession, protein sequence, gene name, nucleotide sequence, Gene Ontology (GO) terms, NCBI Taxa ID, function description and keywords.

In addition to basic annotations, we further annotated PCD regulators in eight model species, including *H. sapiens*, *M. musculus*, *R. norvegicus*, *D. melanogaster*, *C. elegans*, *S. cerevisiae*, *A. thaliana* and *D. rerio.* The integrated knowledge from 102 additional resources covered 16 aspects: *(i)* genetic variation and mutation, *(ii)* functional annotation, *(iii)* structural annotation, *(iv)* physicochemical property, *(v)* functional domain, *(vi)* PTM, *(vii)* disease-associated information, *(viii)* PPI, *(ix)* drug-target relation, *(x)* orthologous information, *(xi)* biological pathway, *(xii)* transcriptional regulator, *(xiii)* mRNA expression, *(xiv)* protein expression/proteomics, *(xv)* subcellular localization and *(xvi)* DNA and RNA element. (Figure 1A, Table S4). These resources were carefully processed and integrated for 17,768 PCD regulators, and a detailed description is presented in the Supplementary Methods. All datasets and annotations in iPCD can be obtained at http://ipcd.biocuckoo.cn/Download.php, accessed on 25 June 2021.

### 3.4. A Data Statistic of PCD Proteins in iPCD

**Figure 2:**
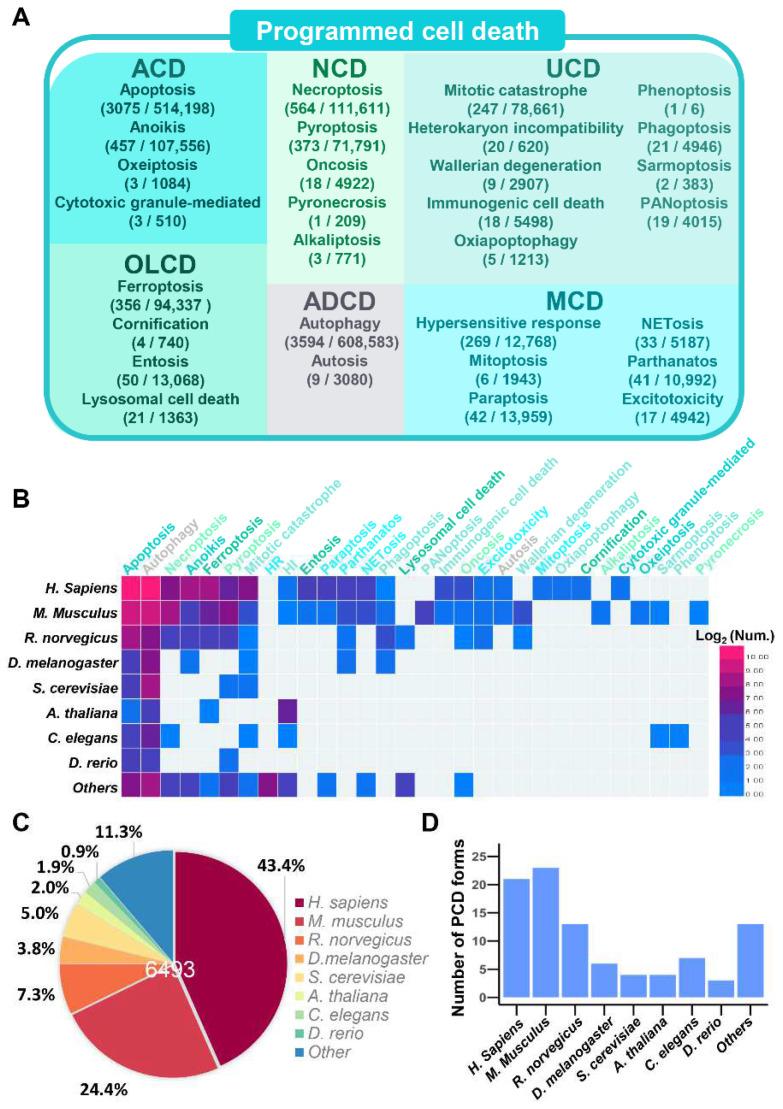
The data statistics of PCD regulators in the iPCD. (**A**) The numbers of PCD regulations and computationally identified PCD regulators are shown according to classification. (**B**) The distribution of experimentally identified PCD regulators in 8 model organisms. (**C**) The percentage distribution of experimentally identified PCD regulators in 8 model organisms. (**D**) The numbers of PCD forms in 8 model organisms.

The iPCD collected 6493 experimentally identified PCD regulators (‘Reviewed’), involved in 30 forms of PCD, and we further conducted an orthologous search to computationally identify 1,091,014 PCD regulators (‘Unreviewed’) in 562 eukaryotes, including 266 vertebrates, 94 plants, 113 metazoa, 33 protists and 56 fungi. The detailed statistics of regulatory proteins in 562 species for 30 PCD forms are presented in Table S5. Not surprisingly, most PCD regulators were observed in vertebrates, especially in humans, which contained 3516 proteins. Similarly, other species have been involved in most forms of PCD, which suggests the relative conservation of PCD in eukaryotes, according to Table S5. In addition, the numbers of experimentally identified positive or negative PCD regulations and computationally identified PCD regulators were shown for each PCD form (Figure 2A). As the best-known three typical PCDs, autophagic cell death/autophagy, apoptosis and necroptosis/necrosis [69] were associated with large numbers of PCD regulators and participate in 3594 (38.7%), 3075 (33.1%) and 564 (6.1%) PCD regulations, respectively. Notably, ferroptosis, which has received widespread attention in recent years, was reported to be involved in 356 (3.8%) regulations. Moreover, the distribution of 30 forms of PCD regulators for eight model organisms was illustrated by Heat map Illustrator (HemI) [91] (Figure 2B), and the detailed data are presented in Table S6. Remarkably, there were 5763 (88.7%) PCD regulators in eight model organisms, and the most predominant species were human and mouse, which encompassed 2818 (43.4%) and 1584 (24.4%) of the known regulators (Figure 2C) and were involved in 21 and 23 PCD forms, respectively (Figure 2D).

**Figure 3:**
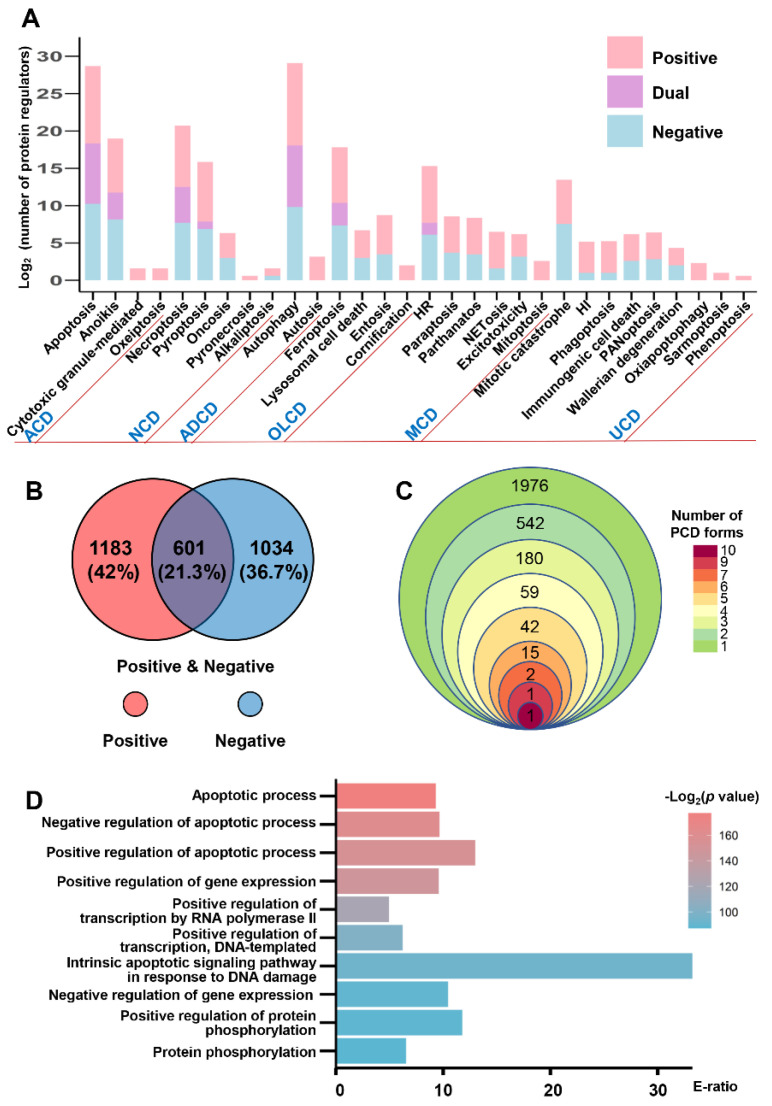
The data counts and analysis of PCD regulators in the iPCD. (**A**) The numbers of positive regulators (‘+’), negative regulators (‘−’) and dual regulators (‘+/−’) are separately presented for each PCD form. (**B**) The overlap of two types human PCD regulators. (**C**) The numbers of PCD regulators involved in different PCD forms. (**D**) The GO-based enrichment analysis of regulators involved in more than 2 PCD processes.

For each regulator annotated with ‘+’ or ‘−’ as positive or negative regulation in PCD based on experimental evidence, the regulatory roles of proteins were counted and demonstrated for each PCD process (Figure 3A). Among them, the distribution of human PCD regulators is shown separately in Figure 3B, which contains 1183 positive regulators, 1034 negative regulators and 601 dual regulators. Then, whether the human regulators were involved in multiple forms of PCD was analyzed and counted (Figure 3C, Table S7). The results showed that 1976 regulators only involved a PCD form, and 842 regulators were involved in more than one PCD process. Since 300 regulators play pivotal roles in the processes of more than two PCD forms, a GO-based enrichment analysis was then performed with these proteins, and various biological processes were uncovered, including apoptotic process, gene expression, DNA or RNA transcription and protein phosphorylation (*p*-value < 10^−26^), which provided definite proof for the functional importance of these PCD protein regulators (Figure 3D).

### 3.5. The Usage of iPCD

**Figure 4:**
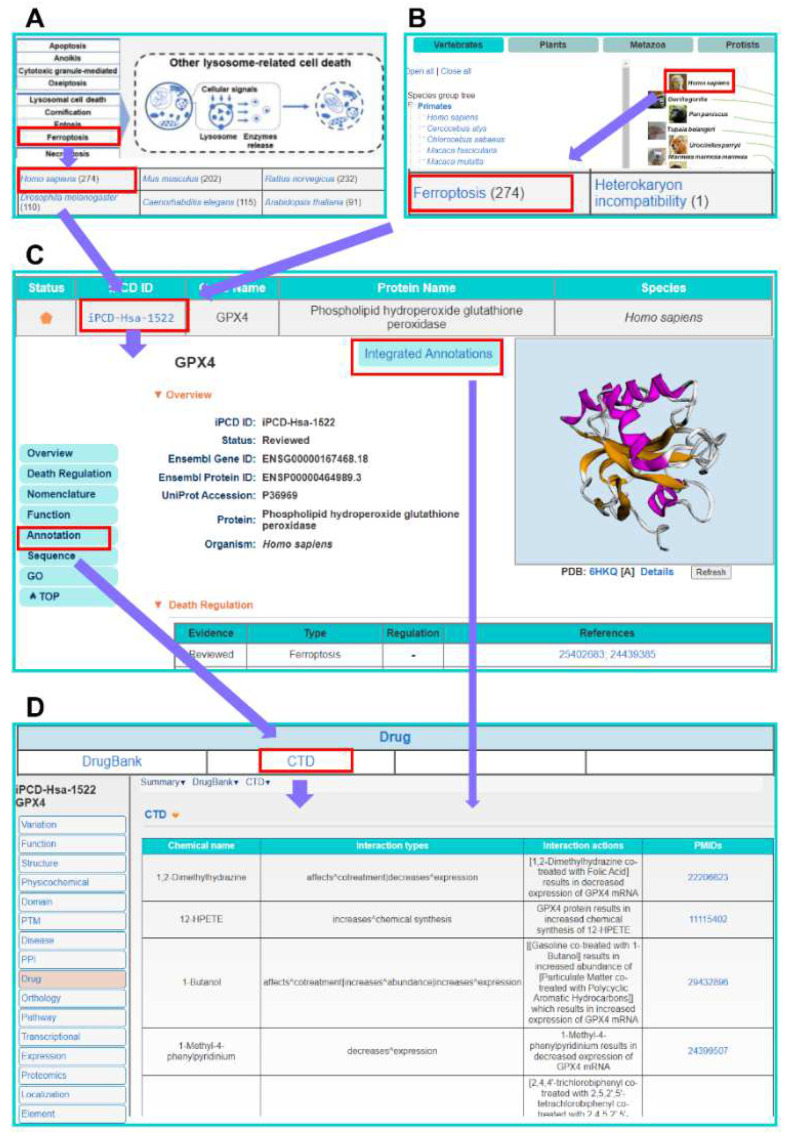
The usage of browse options in the iPCD. (**A**) Browse by process. (**B**) Browse by species. (**C**) The basic information page of human GPX4. Additional annotations could be viewed by clicking the ‘Annotation’ or ‘Integrated Annotations’. (**D**) The additional annotations page of human GPX4 covered 16 aspects. The detailed PTM information of PLMD about GPX4 is shown.

For convenience, the online service of the iPCD database was developed in an easy-to-use manner. Here, human GPX4 protein, the key molecule of ferroptosis, can inhibit ferroptosis by converting lipid hydroperoxides [92] and was selected as an example to describe the usage of iPCD. We provided two options to browse the iPCD database, ‘Browse by process’ or ‘Browse by species’, which can be seen intuitively on the browse page (Figure 4A,B). For browsing by process, users can click ‘Ferroptosis’ and choose the ‘Homo sapiens’ button (Figure 4A), and then by clicking ‘iPCD-Hsa-1522’, as the iPCD ID of human GPX4, the detailed information page of human GPX4 protein can be viewed (Figure 4C). For browsing by species, users can click ‘Homo sapiens’ and choose ‘Ferroptosis’ (Figure 4B), and then the same page of GPX4 can be viewed by clicking ‘iPCD-Hsa-1522’. On this page, basic information is presented, such as Ensembl Gene/Protein ID, UniProt Accession, Organism, Death Regulation, Function description and Protein/Nucleotide Sequence (Figure 4C). More annotations can be accessed by clicking on either ‘Annotation’ in the left navigation bar or ‘Integrated Annotations’ on the middle button. Then, the annotation database summary is presented, and the detailed information can be obtained by clicking the corresponding name (Figure 4C). For example, users can view all PTM information about the GPX4 gene by clicking ‘PLMD’ under the PTM section (Figure 4D). In addition, iPCD provided multiple search options to help users more conveniently access the required information on the search page, including ‘Simple Search’, ‘Advanced Search’, ‘Batch Search’ and ‘BLAST Search’ (Figure S2).

**Figure 5:**
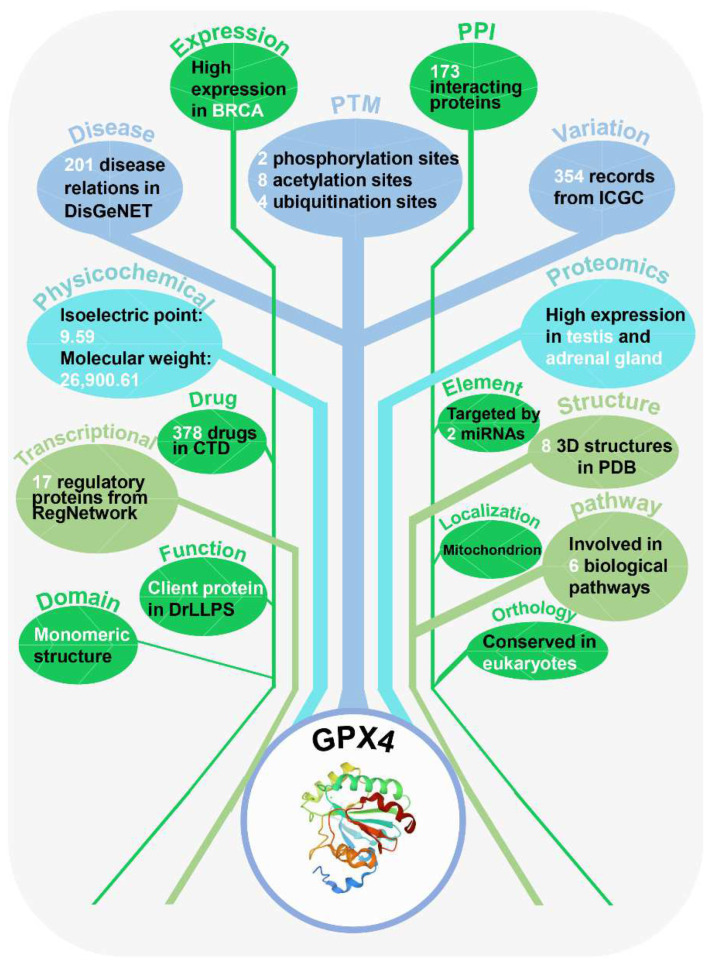
Overview of the 16 aspect annotations information for human GPX4 in the iPCD. The detailed processing for each database is presented in Supplementary Methods, respectively.

The additional annotations of human GPX4 protein contain 16 aspects, which are shown in Figure 5. From the aspect of PPI, 333 information entries of PPI were obtained from seven databases, and 173 proteins were found to potentially interact with GPX4. In terms of biological function, GPX4 is involved in six biological pathways based on KEGG [93] and Reactome [94], such as glutathione metabolism, metabolic pathways and ferroptosis. In particular, GPX4 protein not only inhibits ferroptosis [95], but is also regarded as a client protein that potentially interacts with scaffold proteins in phase separation [96]. Through consulting disease-associated information and combined drug–target relation, an effective experimental strategy may be quickly found. From the DrugBank database, we found that the small molecule drug glutathione is a cofactor for the enzyme glutathione peroxidase and participates in hepatic biotransformation and detoxification process [97]. In addition, 378 types of GPX4-related drug information can also be accessed through the CTD database [98]. Furthermore, the potential roles of the GPX4 protein in 201 diseases were also presented by integrating the DisGeNET database [99]. Associated with mRNA and protein expression in cancer, 11,170 entries of mRNA expression from ICGC in seven cancer types were recorded for GPX4, which is highly expressed in breast cancer [100]. Additionally, the expression level of GPX4 protein was shown in 30 tissues/cells from HPM [101], and it is obvious that GPX4 protein is highly expressed in the testis and adrenal gland. For the annotations of physicochemical properties and functional domain, the isoelectric point (9.59) and molecular weight (26,900.61 Da) of GPX4 were calculated by Compute pI/Mw [102]. The functional domain of GPX4 was annotated by four databases, including Pfam [103], PROSITE [104], InterPro [105] and PIRSF [106]. Additionally, GPX4 was found to have a monomeric structure by accessing Pfam. In parallel, GPX4-associated PTM events were collected from four public resources, including two phosphorylation sites, eight acetylation sites and four ubiquitination sites. Meanwhile, GPX4 can be targeted by hsa-miR-26b-5p and hsa-miR-124-3p according to miRTarBase [107]. Moreover, GPX4 is known to be regulated by 11 proteins and 6 miRNAs, which was presented by RegNetwork [108]. As a non-translocating protein, GPX4 is mainly located in the mitochondria. The annotation information of genetic variation and mutation, structure and orthologues of GPX4 is also shown in Figure 5.

## 4. Discussion

PCD is a precise, genetically controlled biological process of cell suicide that commonly occurs in mature organisms and aims to remove damaged and unnecessary cells or prevent cancer by eliminating nascent neoplastic cells [1]. Research on PCD has been a hotspot since the revelation of its controllability and association with various diseases for decades. Apoptosis, as the first known PCD, is utilized efficiently to induce cancer cell death by chemotherapeutic targeting agents and radiation [109,110,111]. Later, with the emergence of different forms of PCD, a large number of targeted agents were developed for the treatment of tumors. For instance, parkin has been reported to prevent cancer through the inhibition of necroptosis [112], and ferroptosis induced by erastin could kill tumors [113]. Meanwhile, sorafenib was used for the treatment of hepatocellular carcinoma by causing pyroptosis [114]. In the recent past, a number of studies confirmed that different forms of cell death do not seem to have isolated and unique roles but rather act synergistically in multiple diseases [115,116,117]. Undoubtedly, a comprehensive database to collect, verify, integrate and annotate experimentally identified PCD proteins will provide a valuable resource library for better understanding the operation mechanism of cell death to choose the best way to eliminate diseases.

With the in-depth study of PCD, a large number of regulators have been discovered. However, it was always a great challenge to collect, integrate and annotate these PCD-related proteins until Doctor et al. established the first database of apoptosis [85]. Since then, other PCD databases have been developed. The first autophagy database, Autophagy Database, contains 52,021 proteins from 41 species [24]. The first mitotic catastrophe database, MCDB, maintains 188 experimentally identified mitotic catastrophe association proteins and further predicts 1026 proteins [30]. Additionally, the first proteomic database of multiple forms of PCD, CDP, collects 3667 proteins from eight forms of PCD proteomics data [29]. Later, DeepPhagy was constructed as a convenient tool to analyze the autophagic phenotypes of *S. cerevisiae* [89], and fluorescent images were also integrated into iPCD for 35 *ATG* mutants (Figure S3). In contrast, iPCD is comprehensive PCD database, containing 30 forms of PCD and 1,091,014 proteins from 562 eukaryotes, and 102 public resources were integrated for the systematic annotations of 17,768 proteins in eight model organisms. Compared with other PCD databases, the iPCD has a total data size of 125 GB, at least a 600-fold increase in data volume.

Recently, Tsvetkov et al. discovered a novel form of copper-induced PCD, named ‘cuproptosis’, which is dependent on mitochondrial respiration. FDX1 and protein lipoylation play critical roles in cuproptosis [118]. In this process, lipoylated tricarboxylic acid cycle proteins are directly targeted by copper, resulting in proteotoxic stress and subsequent cell death. The regulators of cuproptosis were collected, annotated and orthologously searched in the iPCD updated version 1.1. The conservation of 12 cuproptosis regulators was analyzed in eight model organisms, as shown in Figure S4. In the future, we will continue to maintain and update the iPCD database to incorporate newly identified PCD regulators, and more PCD forms and species will be added to the iPCD. Certainly, more annotations of regulators from additional public resources will be included to provide useful information for users. Taken together, we believe that the iPCD will be a highly useful public resource to analyze PCD.

## Data Availability

All the collected and predicted PCD proteins and multi-layer annotations are freely available at http://ipcd.biocuckoo.cn/Download.php/, accessed on 25 June 2021. The PCD proteins datasets can be downloaded in three data types, including the total dataset, the different PCD forms datasets and eight model species and other species datasets. The annotation datasets can be downloaded by the functional categories of 102 resources, and users can choose the corresponding options according to practical purposes.

## Supplementary Materials

The following supporting information can be downloaded at: https://www.mdpi.com/article/10.3390/cells11132018/s1, Figure S1: The numbers of PCD types and species in iPCD and 12 databases. The detailed information of 12 databases are presented in the Table S1; Figure S1: The four search options of iPCD, including (A) ‘Simple Search’, can show specific information of a protein by inputting a keyword, (B) ‘Advanced Search’ allows to find more information with multiple terms, (C) ‘Batch Search’ with a list keywords in a line-by-line format and (D) ‘BLAST Search’ using a protein sequence in FASTA format; Figure S3: The fluorescence images of *S. cerevisiae* for 35 ATG mutants can be download by clicking on the ‘DeepPhagy’ button or clicking on the corresponding atgs section; Figure S4: The potential orthologs of 12 cuproptosis regulators were computationally identified in 8 model organisms. The existence of cuproptosis potential regulators are marked with a green ball; Table S1: A brief summary of 12 PCD resources. a. The criteria or methods used for data collection, including literature curation, taking from proteomic profiling analyses (proteomic profiling), integration from other public databases (database integration) and computational prediction; Table S2: The distribution of numbers of experimentally identified PCD regulators, which were newly collected into iPCD and not covered by other public PCD resources; Table S3: The detailed summary of 30 PCD forms; Table S4: The 102 public resources of 16 annotation aspects; Table S5: The distribution of regulatory proteins and corresponding PCD forms in 562 eukaryotes; Table S6: The data statistics of experimentally identified proteins for 30 PCD forms in 8 model organisms; Table S7: The data statistics of human regulators involved in different forms of PCD.
