# Supplementary material for: iPCD: A Comprehensive Data Resource of Regulatory Proteins in Programmed Cell Death
Source: Cells. 2022 Jun 24;11(13):2018. doi: 10.3390/cells11132018 (PMC9265749; doi:10.3390/cells11132018)
Supplement: Supplementary file 1 [file cells-11-02018-s001.zip › cells-1728692-supplementary.pdf]

Supplementary Data:

iPCD: A Comprehensive Data Resource of Regulatory Proteins in Programmed Cell Death

Dachao Tang, Cheng Han, Shaofeng Lin, Xiaodan Tan, Weizhi Zhang, Di Peng, Chenwei Wang and Yu Xue

Running title: *A database of programmed cell death regulators*

Corresponding author: Yu Xue, Department of Bioinformatics and Systems Biology, MOE Key Laboratory of Molecular Biophysics, Hubei Bioinformatics and Molecular Imaging Key Laboratory, Center for Artificial Intelligence Biology, College of Life Science and Technology, Huazhong University of Science and Technology, Wuhan 430074, China; Nanjing University Institute of Artificial Intelligence Biomedicine, Nanjing, 210031, China. Tel: +86-27-87793903; Fax: +86-27-87793172; E-mail: xueyu@hust.edu.cn. Chenwei Wang, E-mail: wangchenwei@hust.edu.cn.
The first two authors contributed equally to this work.

Supplementary Data Index

Supplementary methods 3
Supplementary references 37
Supplementary Figure S1 43
Supplementary Figure S2 44
Supplementary Figure S3 45
Supplementary Figure S4 46
Supplementary Tables 47

Supplementary methods

The iPCD database was implemented in JavaScript, PHP and MySQL. We tested the online service of iPCD on a number of mainstream internet browsers, including Internet Explorer (IE) 20H2, Mozilla Firefox 98.0.1, 360 Browser SE13.1, Google Chrome 99, Microsoft Edge 98 under Windows 10 Operating Systems and Safari 14.1.1 of Apple Mac.

Besides the manual collection of experimentally identified PCD proteins for 287 species from PubMed, iPCD also integrated knowledge from 113 existing resources, including 11 PCD databases (Supplementary Table S1) and 102 additional public resources (Supplementary Table S4). 17,768 PCD regulators were annotated with 102 public resources covered 16 aspects in 8 model organisms, including *H. sapiens, M. musculus, R. norvegicus, D. melanogaster, C. elegans, S. cerevisiae, A. thaliana, and D. rerio.* The detailed processing information for each resource are shown below.

1. Programmed cell death (PCD) databases

*1.1) DeathBase (*http://www.deathbase.org/*) [1]*

DeathBase is a well-organized data database of proteins involved in different cell death processes, mainly focuses on apoptosis. The basic information of PCD associated proteins including uniprot id, external id, species, the PCD type, biological pathway and simple protein description in several species. The ‘protein_list.txt’ file was downloaded from the ‘Downloads’ page of Deathbase. This database contains 213 proteins, but we just kept PCD-related proteins by ‘process_id’ entry filtration. The uniprot id, gene name and cell death type of PCD-related proteins was used to re-curated and search from literature by PubMed. We annotated proteins with ‘+’ or ‘-’ as positive and negative PCD regulators, and only positive or negative regulators were reserved. After rigorous and careful screening, we obtained 115 non-redundant PCD-related proteins.

1.2) yApoptosis (http://www.ycelldeath.com/yapoptosis/) [2]

yApoptosis is an extensively-curated yeast apoptosis database, that annotated apoptotic genes with pathway information, GO terms, relevant literature and linked to protein or genes interaction information. yApoptosis structures apoptosis genes interaction network to show the

crosstalk between different genes. The ‘yApoptosis.txt’ was downloaded by clinking the ‘Apoptotic-related genes: Text (tab delimited)’ section from the ‘Download’ page of yApoptosis. 51 apoptotic-related genes or proteins were obtained. To get the necessary information, we used three keys to re-curated and search from PubMed, including gene name, uniprot id and pubmed id. Finally, we integrated 39 apoptosis regulators into iPCD database from yApoptosis after carefully verified.

1.3) Human Autophagy Database (HADb) (http://www.autophagy.lu/index.html) [3]

Human Autophagy Database (HADb) is a human autophagy-dedicated database, that provides human genes or proteins involved directly or indirectly in autophagy. The autophagy-related proteins were annotated with motif, synonym, domains, site and so on. We acquired autophagy-related genes or proteins by the page of ‘Clustering’ from HADb database. Next, we search and downloaded 222 proteins information from Uniprot database by using the gene id of HADb database. Then, the careful identification and annotation (‘+’ or ‘-’) was carried out by searching the PubMed database. At last, we obtained 197 autophagy regulators from Human Autophagy Database.

1.4) Autophagy Database (http://autophagy.info/) [4]

Autophagy Database is first developed autophagy database, that provides up-to-data relevant information, including functional and structural information on a multitude of autophagy-related proteins. The Autophagy Database was updated at 2017, including 582 reviewed autophagy-related proteins and 51,439 homologous in multiple species. The ‘autophagyDB.tar.gz’ was downloaded from the ‘Download’ page of Autophagy Database. The ‘atg_genes_detail.dat’ were used to obtain 582 reviewed proteins, those proteins were filtered out by the ‘status_id’. Due to the reviewed proteins was labeled as ‘1’, 582 autophagy-related proteins were obtained. Then, the gene ID was converted to the protein ID by Uniprot database, and these proteins were re-identified by search PubMed. Finally, 365 autophagy positive or negative regulators were integrated into iPCD.

1.5) Autophagy Regulatory Network (ARN) (http://autophagyregulation.org/) [5]

Autophagy Regulator Network (ARN) database is an excellent resource to constructed the

autophagy proteins regulatory network. ARN integrates known autophagy protein regulators and their interactions, predicts novel autophagy regulators and possible transcriptional and post-transcriptional regulators, contains 14,018 proteins, 386 miRNAs, and 397,764 interactions. The 'autophagy_proteins_and_direct_regulators_of_autophagy_proteins_arn.tsv' file was downloaded from the 'download' page of ARN database (version: 1.0.6). The 'source_name' and 'source_uniprotAC' were used to re-curated and annotated ('+' or '-') from the PubMed's literature. In the end, 97 autophagy regulators were collected and integrated into iPCD.

1.6) Human Autophagy Modulator Database (HAMdb) (http://hamdb.scbdd.com) [6]

Human Autophagy Modulator Database (HAMdb) is a comprehensive database that contains autophagy-related autophagy, chemicals and microRNAs, related biological pathway, disease information and drug target. HAMdb contains 796 autophagy-related proteins, 841 chemical reagent and 132 autophagy-related microRNAs. The 'protein-role.xlsx' file was downloaded from the HAMdb database. We combined gene names, species and corresponding PubMed ID information to re-check and map uniprot ID. Then, these proteins were classified as positive or negative regulators by reviewing the content of the article from PubMed. In total, 608 autophagy regulators were included into iPCD.

1.7) Autophagy and Tumor Database (ATdb) (http://www.bigzju.com/ATdb/#/) [7]

Autophagy and Tumor Database (ATdb) is novel database that integrates the connection between autophagy and tumor. ATdb connects 25 types of cancers with 137 autophagy-related genes. The 'included_genes.txt' was downloaded from the ATdb database. The gene ID and gene name were used as keywords to download necessary information of proteins from uniprot database. Then, 136 proteins were re-identified and screened from the literatures of PubMed. After careful curation, these proteins were all identified as autophagy regulators (positive or negative) and were included in the iPCD database.

1.8) FerrDb (http://www.zhounan.org/ferrdb/) [8]

FerrDb is the first ferroptosis database that dedicates to ferroptosis regulators and ferroptosis-disease associations. FerrDb consists of six independent data sets, including

ferroptosis driver, ferroptosis suppressor, ferroptosis marker, chemical inducer regent of ferroptosis, chemical inhibitor regent of ferroptosis and ferroptosis-related disease. The '1_info_driver.xlsx' and '1_info_suppressor.xlsx' two files were downloaded from the 'Download' page of FerrDb. The protein names, species, uniprot ID and PMID were conserved and re-curated from the PubMed. At last, 148 positive regulators and 57 negative regulators were included into iPCD database.

1.9) MCDB (http://www.combio-lezhang.online/MCDB/index_html/) [9]

Mitotic Catastrophe Database (MCDB) is the first mitotic catastrophe database that provides comprehensive information about mitotic catastrophe-related genes or proteins, chemical interventions. In addition, extended functions were also provided, such as protein sequence similarity comparison and target prediction rested on compound structure similarity. The 'MC-related gene and protein' file was downloaded from the MCDB database. We only collected experimentally identified proteins by the 'MC-PMID' section filtration, 186 proteins were obtained. These proteins were re-identified by carefully reading abstract or full text of papers from PubMed. Finally, 169 mitotic catastrophe regulators were curated and conserved.

1.10) Cell Death Proteomics database (CDP) (http://celldeathproteomics.uio.no/) [10]

Cell Death Proteomics database (CDP) is a cell death proteomics database that involves 8 types programmed cell death proteomics, including apoptosis, autophagy, cytotoxic granule-mediated cell death, excitotoxicity, mitotic catastrophe, paraptosis, pyroptosis, and Wallerian degeneration. The CDP database contains more than 6500 records and 3700 proteins, that were annotated with UniProt-KB and gene ontology (GO). The 'cell_death_database.tsv' file were downloaded from the CDP database. The 'uniprot_genename' and 'uniprot_ac' were conserved and re-identified. Due to these proteins were the results of analysis of different cell death proteomics data, there is no experimental evidence for these proteins. But, we also revalidated and reclassified these proteins according to the corresponding published literature from PubMed in order to avoid missing proteins as much as possible. In the end. 599 proteins were re-identified as PCD positive or negative regulators, and were included into iPCD database.

1.11) ncRDeathDB (http://www.rna-society.org/ncrdeathdb/) [11]

ncRDeathDb is a Non-coding RNAs (ncRNAs) database that integrated miRNA, IncRNAs and snoRNAs related three types of programmed cell death, including apoptosis, autophagy and necrosis. The ncRDeathDb were developed based on the miRDeathDB (http://rna-world.org/mirdeathdb/), that contains 2403 apoptosis-associated entries, 2205 autophagy-associated entries and 7 necrosis-associated entries. The 'allNcRNACelldeathData.txt' file was downloaded by the 'Download & API' page of ncRDeathDB. The 'Gene_Symbol', 'Pathway', 'PMID' and 'geneid' were conserved and re-identified from the literature of PubMed. In total, 419 proteins were identified as the PCD regulators ('+' or '-') and included into iPCD.

2. Genetic variation & mutation

2.1) TCGA (https://www.cancer.gov/tcga) [12]

The TCGA mutations related information of 36 available disease were downloaded by BROAD Institute (http://gdac.broadinstitute.org/runs/stddata__2016_01_28/data, Oncotated calls, level 3), including ACC (adrenocortical carcinoma), BLCA (bladder urothelial carcinoma), BRCA (Breast invasive carcinoma), COAD (colon adenocarcinoma), CESC (cervical squamous cell carcinoma and endocervical adenocarcinoma), CHOL (cholangiocarcinoma), COADREAD (colorectal cancer), DLBC (lymphoid neoplasm diffuse large B-cell lymphoma), ESCA (esophageal carcinoma), GBM (glioblastoma multiforme), GBMLGG (glioma), HNSC (head and neck squamous cell carcinoma), KICH (kidney chromophobe), KIPAN (pan-kidney cohort), KIRC (kidney renal clear cell carcinoma), KIRP (kidney renal papillary cell carcinoma), LAML (acute myeloid leukemia), LGG (brain lower grade glioma), LIHC (liver hepatocellular carcinoma), LUAD (lung adenocarcinoma), LUSC (lung squamous cell carcinoma), OV (ovarian serous cystadenocarcinoma), PAAD (pancreatic adenocarcinoma), PCPG (pheochromocytoma and paraganglioma), PRAD (prostate adenocarcinoma), SKCM (skin cutaneous melanoma), READ (rectum adenocarcinoma), SARC (sarcoma), STAD (stomach adenocarcinoma), STES (stomach and esophageal carcinoma), TGCT (testicular germ cell tumors), THCA (thyroid carcinoma), THYM (thymoma), UCEC (uterine corpus endometrial carcinoma), UCS (uterine carcinosarcoma), and UVM (uveal melanoma). The UniProt IDs of iPCD were used as indexes to map the data of 36 TCGA mutation file. Of these, the 'PRJ_code', 'Chromosome', 'Start_position', 'End_position', 'Strand', 'Variant_Classification', 'Genome_Change', 'cDNA_Change', 'Codon_Change' and 'Protein_Change' were conserved and

integrated into annotations information of iPCD. In total, 121,420 entries of cancer mutations were obtained.

2.2) ICGC (http://icgc.org/) [13]

All simple somatic mutations data of tumor tissues from ICGC were downloaded (https://dcc.icgc.org/releases/release_28/Projects/, release 28). The column entitled 'gene_affected' (Ensembl gene IDs) were used to map ICGC mutation information. Most of mutation related information of ICGC were integrated, such as 'icgc_mutation_id', 'icgc_donor_id', 'project_code', 'icgc_sample_id', 'matched_icgc_sample_id', 'chromosome', 'matched_icgc_sample_id', 'submitted_sample_id', "chromosome_strand', 'assembly_version', 'mutation_type', 'reference_genome_allele', 'mutated_from_allele' and so on. Here, we obtained 46,553,541 entries of cancer mutations.

2.3) COSMIC (https://cancer.sanger.ac.uk/cosmic) [14]

the 'CosmicMutantExport.tsv.gz' file was downloaded from the download page of COSMIC (https://cancer.sanger.ac.uk/cosmic/download). Ensembl transcript IDs were used as primary accession numbers for mapping cancer mutations data of COSMIC to iPCD. Most of the information of COSMIC was integrated, such as Sample, Mutation, Primary site, Primary histology, Mutation CDS, Mutation AA, Description, Position and Strand and so on. Here, we obtained 2,206,678 entries of cancer mutations from COSMIC database.

2.4) dbSNP (https://www.ncbi.nlm.nih.gov/snp/) [15]

We downloaded 25 reference SNP files of *Homo sapiens* from the ftp site of dbSNP (release Sept, 2018). The uniport IDs were used as the indexes to map the corresponding information of dbSNP. Then, the annotation information of iPCD were generated by match. These columns including 'iPCD ID', 'UniProt', 'Link', 'dbSNP', 'Position', 'AA residue', 'Type', 'Position', 'Alleles', and 'Frequency'. Finally, 1,419,429 entries were obtained and integrated into annotations of iPCD.

2.5) IntOGen (https://www.intogen.org/) [16]

We downloaded the 'intogen_driver_mutations_catalog-2016.5.zip' file from the 'Downloads'

page of IntOGen. The Ensembl transcript IDs were used as match indexes for mapping cancer mutations information to iPCD. The annotation file of iPCD was generated, the specific columns of the annotation file included ‘iPCD ID’, ‘Ensembl transcript’, ‘position’, ‘protein change’, ‘cancer’, ‘consequence’, ‘gDNA’, ‘cDNA’ and ‘Protein’. After integrated, we obtained 101,817 entries about cancer mutation from IntOGen database.

2.6) MIMP (http://mimp.baderlab.org/) [17]

We downloaded the ‘tcga_rewiring_events_prob.tab’ file from the ‘Download’ page of MIMP (in Jun, 2021). The human gene names (the column entitled ‘gene’) were used as the primary accession numbers to map cancer mutations information of MIMP to iPCD. Finally, the annotation file of iPCD about the MIMP owned mutations information was generated, these columns entitled as ‘iPCD ID’, ‘RefSeq Nucleotide’, ‘Position’, ‘Score (WT)’, ‘Score (MT)’, ‘Pwm’, ‘Effect’ and ‘Log (Ratio)’, respectively. Here, we obtained 8528 entries of cancer mutation from MIMP.

2.7) VarCards (http://varcards.biols.ac.cn/) [18]

All SNP files were downloaded from the download page of Varcards (http://varcards.biols.ac.cn/, in Oct 2020). The Ensembl gene IDs were used as the primary accession numbers to map cancer mutations of VarCards to iPCD. After integrated, these annotation columns information included ‘iPCD ID’, ‘Ensembl’, ‘Chromosome’, ‘Position’, ‘Ref’, ‘Alt’, ‘Exon’, ‘cDNA’ and ‘protein’. In total, 128,816,513 entries of cancer mutation information of VarCards were integrated into iPCD.

3. Functional annotation

3.1) DrLLPS (http://llps.biocuckoo.cn/) [19]

DrLLPS is integrated database about liquid-liquid phase separation, which contains 437,887 known and potential phase separation related proteins. The data were downloaded from the download page of DrLLPS (in May 2021). The Ensembl IDs were used as the primary accession numbers to match the functional information of DrLLPS to iPCD. Finally, we conserved ‘Ensembl’, ‘Link’, ‘DrLLPS ID’, ‘LLPS Type’, ‘Condensate’ and ‘Source’ in the annotation file of iPCD. Here, we obtained 6,280 entries of function information from DrLLPS.

3.2) iEKPD (http://iekpd.biocuckoo.org/) [20]

Previously, we developed an updated database iEKPD 2.0, which contained 197,348 phosphorylation regulators, including 109,912 protein kinases, 23,294 protein phosphatases and 68,748 PPBD (proteins containing phosphoprotein-binding domain) containing proteins in 164 eukaryotic species. The data were downloaded from the download page of iEKPD. UniProt IDs were used as the match indexes to map the iEKPD function information to iPCD. Annotation columns included 'iPCD ID', 'UniProt', 'Link', 'ID', and 'Family'. In the end, we obtained 2,400 entries of function information from iEKPD.

3.3) iUUCD (http://iuucd.biocuckoo.org/) [21]

In 2018, we developed an updated database iUUCD 2.0, which contained 136,512 UB/UBL (ubiquitin and ubiquitin-like (UB/UBL) conjugations) regulators, including 1230 E1s (ubiquitin-activating enzymes), 5636 E2s (ubiquitin-conjugating enzymes), 93,343 E3s (ubiquitin-protein ligases), 9548 DUBs (deubiquitinating enzymes), 30,173 UBDs (ubiquitin/ubiquitin-like binding domains) and 11,099 ULDs (ubiquitin-like domains) in 148 eukaryotic species. The corresponding data was downloaded from the iUUCD. The uniprot id were used as the primary accession numbers to map the function information of iUUCD to iPCD. The annotation columns included 'iPCD ID', 'UniProt', 'ID' and 'Family'. After integrated. iPCD contained 1693 entries of function information from iUUCD.

3.4) WERAM (http://weram.biocuckoo.org/) [22]

In 2017, we developed a database WERAM, which contained over 580 experimentally identified histone regulators and ~900 site-specific regulator-histone relations from 8 species, in total more than 20 thousand non-redundant histone regulators from 148 eukaryotes. The corresponding data was downloaded from the WERAM. The gene names were used as the primary accession numbers to map the function information of WERAM to iPCD. Similarly, the annotation information contained 'iPCD ID', 'UniProt', 'ID' and 'Family'. In the end, we acquired 319 entries of function information from the WERAM.

3.5) AnimalTFDB (http://bioinfo.life.hust.edu.cn/AnimalTFDB/) [23]

We downloaded the TFs (transcription factors) and TF cofactors (transcription cofactors) files of 5 species (*H. sapiens, M. musculus, R. norvegicus, D. melanogaster and C. elegans)* from the 'Download' page of AnimalTFDB (http://bioinfo.life.hust.edu.cn/AnimalTFDB/#!/download, Version 3.0, in Nov, 2020). In this work, Ensembl gene IDs were used as the match indexes to map corresponding information from AnimalTFDB. Enseml id, link, AnimalTFDB's annotation and family were included into iPCD. After integrated, we acquired 2,926 entries from AnimalTFDB.

3.6) PlantTFDB (http://planttfdb.cbi.pku.edu.cn/) [24]

The 'Ath_TF_list.txt.gz' files were downloaded from the 'Download' page of PlantTFDB (http://planttfdb.cbi.pku.edu.cn/download.php, Version 4.0, in Nov 2020). The TF families in the column 'Family' were conserved, Ensembl gene IDs in the column 'Gene_ID' were used as match indexes to map corresponding information of PlantTFDB' to iPCD. In addition, the link of PlantTFDB for each TF was also added into the annotations of iPCD. After integrated, we acquired 70 entries information of *A. thaliana* from the PlantTFDB.

3.7) HAMAP (http://hamap.expasy.org/) [25]

In this study, the 'rules_index.dat' file and the 'hamap_alignments' file were downloaded from the FTP server (ftp://ftp.expasy.org/databases/hamap/) of HAMAP (release 2020_05, in Oct 2020). The UniProt IDs were used as the primary accession numbers to map corresponding information from the 'hamap_alignments' file of HAMAP, and the HAMAP id, identifier and description were extracted from the file 'rules_index.dat'. in the end, 168 entries of HAMAP were integrated into iPCD.

3.8) neXtProt (https://www.nextprot.org/) [26]

We downloaded the 'nextprot_ac_list_all.txt' file from the FTP server of neXtProt (ftp://ftp.nextprot.org/pub/current_release/ac_lists/, in Nov 2020). The UniProt IDs were used as the match indexes to map corresponding information from the neXtProt. neXtProt IDs were extracted and the link of neXtProt for each entry was added into the annotations of iPCD. At last 3,488 human annotated proteins were integrated from neXtProt.

3.9) CGDB (http://cgdb.biocuckoo.org/) [27]

In 2017, we developed a database CGDB, which contained ~73,000 circadian-related genes in 148 eukaryotes. In this work, the gene names were used to map function information of CGDB to iPCD. The UniProt ID, CGDB link, and CGDB ID were integrated into iPCD. Here, we obtained 72,800 CGDB proteins and corresponding information.

3.10) MultitaskProtDB-II (http://wallace.uab.es/multitaskII/) [28]

The proteins information of multitasking was exported from the 'DataBase' page of MultitaskProtDB-II (http://wallace.uab.es/multitaskII/proteins_list.php, in Oct 2020). The UniProt IDs were used as the match indexes to map information from MultitaskProtDB-II. The annotation columns of iPCD included 'iPCD ID', 'UniProt', 'Link', 'Protein name', 'Canonical Function', 'Moonlighting Function' and 'PMIDs'. After integrated, we acquired 126 protein annotation entries.

3.11) MoonDB (http://moondb.hb.univ-amu.fr/) [29]

We downloaded the 'all_EMF_annotation.tsv' file from the 'Downloads' page of MoonDB (http://moondb.hb.univ-amu.fr/downloads, Version 2.0, in Oct 2020). The UniProt IDs were mapped to iPCD. The annotation columns of iPCD included 'iPCD ID', 'UniProt', 'Link', 'GO ID 1', 'Function 1150', 'DO ID 2', 'Function', 'Association probability' and 'Interaction probability'. In the end, 445 proteins of MonnDB were integrated into iPCD.

3.12) CORUM (http://mips.helmholtz-muenchen.de/corum/) [30]

We downloaded the 'allComplexes.txt' file from the CORUM (http://mips.helmholtz-muenchen.de/corum/#download, in Oct 2020). The UniProt IDs were used as the indexes to map the corresponding information of CORUM. Finally, the annotations were integrated, including 'Complex name', 'Cell line', 'Other subunits' and 'PMIDs'. Here, 6591 entries were obtained from the CORUM.

3.13) CellMarker (http://biocc.hrbmu.edu.cn/CellMarker/) [31]

We downloaded the ‘all_cell_markers.txt’ file from the ‘Download’ page of CellMarker (http://biocc.hrbmu.edu.cn/CellMarker/download.jsp, in Nov 2020). The UniProt IDs were used as indexes to map function information from CellMarker to iPCD. The integrated Columns included ‘Tissue type’, ‘Cancer type’, ‘Cell type’, ‘Cell name’, ‘Markers’, ‘Source’ and ‘PMIDs’. After integrated, 16,829 cell markers were obtained from CellMarker.

3.14) GPCRdb (http://www.gpcrdb.org/) [32]

The ‘uniprot_mapping.txt’ file was downloaded from the ‘Tutorial’ page of GPCRdb (http://docs.gpcrdb.org/linking.html, in May 2021). The UniProt IDs were extracted and used to map corresponding information of GPCRdb to iPCD. The UniProt ID and GPCRdb ID were conserved. In the end, 221 proteins and corresponding annotation were integrated into iPCD.

3.15) EuRBPDB (http://EuRBPDB.syshospital.org/) [33]

The ‘Homo_sapiens.RBP.txt.gz’, ‘Rattus_norvegicus.RBP.txt.gz’, ‘Danio_rerio.RBP.txt.gz’, ‘Caenorhabditis_elegans.RBP.txt.gz’ and ‘Drosophila_melanogaster.RBP.txt.gz’ five files were downloaded from the download website (http://eurbpdb.syshospital.org/download.html, in May 2021) of EURBPDB. The Ensembl gene IDs were used as the primary accession numbers to map information of EuRBPDB to iPCD and the RBP type was conserved. After integrated, we obtained 2,214 entries from EuRBPDB.

4. Structural annotation

4.1) PDB (http://www.rcsb.org/) [34]

In this work, all files were downloaded from the FTP server of PDB (ftp://ftp.rcsb.org/pub/pdb/data/structures/divided/pdb/). For each .ent file, the PDB entry, UniProt ID, sequence, and chain were first extracted. And then, the UniProt IDs were used to map PDB information to iPCD. The integrated annotation information included PDB link, PDB ID, description, ‘chain’, ‘start’ and ‘end’. Finally, we obtained 67,819 proteins 3D structures from PDB.

4.2) SCOP2 (http://scop2.mrc-lmb.cam.ac.uk/) [35]

We downloaded ‘scop_fa_represeq_lib20200925.fa’, ‘scop-cla-latest.txt’ and ‘scopdes-

latest.txt' three files from download page of SCOP2 (http://scop2.mrc-lmb.cam.ac.uk/downloads/, in Nov 2020). In this work, the UniProt IDs were used as the keys to map corresponding information of SCOP2 to iPCD. The final annotation columns included UniProt ID, SCOP2 link, SCOP2 ID, domain and domain information. After integrated, we obtained 3071 entries from SCOP2.

4.3) IUPred (https://iupred.elte.hu/) [36]

IUPred is a web server for the prediction of intrinsically unstructured regions of proteins based on estimated energy content. In this work, the program package was downloaded from the 'Downloads' page of IUPred. The IUPred were integrated to predict potentially disordered regions in PCD proteins of 8 model organisms.

4.4) DisProt (https://disprot.org) [37]

In this work, the 'search_in_disprot.tsv' file was downloaded from the download page of DisProt (in Jun 2021). The UniProt IDs were used as the keys to map corresponding information of DisProt to iPCD. The 'DidProt ID', 'Region ID', 'Start', 'End', 'Term Namespace' and 'PMIDs' were conserved. In addition, the DisProt link for each protein was added into the annotation file of iPCD. After integrated, we acquired 1983 proteins of DisProt and corresponding annotation information.

4.5) DNAproDB (https://dnaprodb.usc.edu/) [38]

In this work, the 'dnaprodb_2.1.7z' was downloaded from the download website of DNAproDB (https://dnaprodb.usc.edu/data/dnaprodb_2.1.7z, Version 2.1, in May 2021). The UniProt IDs were used as the keys to map the corresponding information of DNAproDB. The DNAproDB ID and sequence were conserved and DNAproDB link for each protein were added into the annotations of iPCD. Finally, 645 entries were obtained from the DNAproDB.

5. Physicochemical property

5.1) AAindex (http://www.genome.jp/aaindex/) [39]

AAindex is a database of numerical indices representing various physicochemical and biochemical properties of amino acids and pairs of amino acid. We downloaded the 'aaindex1' file from the FTP server of AAindex (ftp://ftp.genome.jp/pub/db/community/aaindex/, in Oct 2020).

Each physicochemical property, scores for 20 types of amino acids were extracted and mapped to iPCD.

5.2) Compute pI/Mw (https://web.expasy.org/compute_pi/) [40]

Compute pI/Mw is a tool, which allows the computation of the theoretical pI (isoelectric point) and Mw (molecular weight) for a list of UniProt Knowledgebase (Swiss-Prot or TrEMBL) entries. The pI and Mw of Compute pI/Mw was adopted for all PCD proteins in 8 model organisms (in Jun, 2021).

6. Functional domain

6.1) Pfam (http://pfam.xfam.org/) [41]

The 'Pfam-A.clans.tsv.gz' and 'PfamA.regions.uniprot.tsv.gz' two files were downloaded from the FTP server (ftp://ftp.ebi.ac.uk/pub/databases/Pfam) of Pfam (in Dec 2020). The UniProt IDs were used as the keys to map information of Pfam to iPCD. The final annotation columns included 'Pfam Link', 'Pfam ID', 'Family', 'Start' and 'End'. After integrated, 39,166 entries were obtained from the Pfam.

6.2) PROSITE (https://prosite.expasy.org/) [42]

In this work, we downloaded the 'prosite_alignments.tar.gz' file from the FTP server (ftp://ftp.expasy.org/databases/prosite/) of PROSITE (in Jan, 2022). The UniProt IDs were used as the keys to map corresponding information to iPCD. For annotation information, the PROSITE link, PROSITE ID and corresponding description were integrated. In the end, 27,787 entries of PROSITE were included in the iPCD.

6.3) InterPro (http://www.ebi.ac.uk/interpro/) [43]

We downloaded the 'protein2ipr.dat.gz' file from the 'Download' page of InterPro (http://www.ebi.ac.uk/interpro/download.html, in Dec 2020). The UniProt IDs were used as the indexes to map necessary information from InterPro to iPCD. The InterPro IDs, UniProt IDs, domain name, start and end positions were conserved. After integrated, 206,446 entries were extracted as the annotations of iPCD from InterPro.

6.4) PIRSF (https://proteininformationresource.org/pirwww/dbinfo/pirsf.shtml) [44]

In this work, we downloaded the 'pirsfinfo.dat' file from the FTP server of PIRSF (ftp://ftp.pir.georgetown.edu/databases/pirsf/, in Dec 2020). Similarly, the UniProt IDs were used as the keys to map corresponding annotations data from PIRSF. For each entry, the PIRSF link, PIRSF ID and corresponding description were integrated into the iPCD. At last, we obtained 8838 entries from PIRSF.

6.5) PRINTS (http://130.88.97.239/PRINTS/index.php) [45]

We downloaded the compressed file named 'prints42_0.dat' from the FTP server of PRINTS (ftp://ftp.ebi.ac.uk/pub/databases/prints/, in Oct 2020). For each entry, the PRINTS IDs, UniProt IDs, identifiers and corresponding description were extracted. Then, the UniProt IDs were used as the keys to acquired corresponding information from the PRINTS. After integrated, 643 proteins and corresponding annotations were obtained.

7. Post-translational modification

7.1) EPSD (http://epsd.biocuckoo.cn/) [46]

In 2021, we developed a database EPSD, which contained 1,616,804 experimentally identified p-sites in 209,326 phosphoproteins across 68 eukaryotic species. The data of EPSD was downloaded from the download page of EPSD website. For each entry, the Uniprot IDs, EPSD IDs, AA, position and PMIDs were extracted and conserved. Similarly, UniProt IDs were used as the keys to map and acquired corresponding annotation from EPSD. After integrated, we obtained 229,470 entries.

7.2) PLMD (http://plmd.biocuckoo.org) [47]

Previously, we developed a database PLMD, which contained 284,780 modification events in 53,501 proteins across 176 eukaryotes. In this work, the Uniprot ID, PLMD link, PLMD ID, position, lysine modification type and PMIDs were extracted from the downloaded data of PLMD. Same as before, the UniProt IDs were used as the keys to match corresponding annotations information from PLMD. After integrated, 63,673 entries were taken from the PLMD.

7.3) dbPTM (http://dbPTM.mbc.nctu.edu.tw/) [48]

The 'dbptm.zip' file was downloaded from the download page of dbPTM (in May 2021). For each entry, the UniProt ID, position, AA, modification type and PMIDs were extracted and conserved. Then, we mapped the corresponding annotations information from the dbPTM by using the UniProt IDs. Finally, we obtained 10,131 annotation entries from the dbPTM.

7.4) PhosphoSitePlus (http://www.phosphosite.org/) [49]

All modification files were downloaded from the download page of PhosphoSitePlus (in Apr, 2021), including 'Acetylation_site_dataset', 'Methylation_site_dataset', 'O-GalNAc_site_dataset', 'O-GlcNAc_site_dataset', 'Phosphorylation_site_dataset', 'Sumoylation_site_dataset', and 'Ubiquitination_site_dataset'. The UniProt IDs were used as the primary accession numbers to map the corresponding annotation information from PhosphoSitePlus to iPCD. the annotation columns contained PhosphoSitePlus link, position, modification type for each entry. After integrated, 146,080 entries were taken from PhosphoSitePlus.

7.5) iPTMnet (http://proteininformationresource.org/iPTMnet) [50]

In this work, we downloaded the 'ptm.xtx' file from the download page of iPTMnet (in May, 2021). Similarly, the UniProt IDs were used as the indexes to map corresponding information from the iPTMnet. For each entry, the iPTMnet link, position, modification type and PMIDs were conserved and integrated into the iPCD. In the end, we acquired 70,542 entries from the iPTMnet.

7.6) HPRD (http://www.hprd.org/) [51]

The 'HPRD_FLAT_FILES_041310.tar.gz' file was downloaded from the HPRD (in Oct, 2020). The compressed file contained 'POST_TRANSLATIONAL_MODIFICATIONS.txt' and 'HPRD_ID_MAPPINGS.txt'. First, the HPRD ID were used to acquire the corresponding UniProt ID for each protein in two files. Then, the HPRD links, positions, AA, modification types and PMIDs were integrated into the iPCD by mapping UniProt IDs. Finally, 19,399 entries were obtained from the HPRD.

8. Disease-associated information

8.1) ClinVar (https://www.ncbi.nlm.nih.gov/clinvar/) [52]

The 'clinvar_20190506.vcf' file was downloaded from the FTP server of ClinVar (ftp://ftp.ncbi.nlm.nih.gov/pub/clinvar/, in Dec, 2020). Ensembl gene IDs were acquired according to the method of EPSD [46]. Then, the UniProt IDs were used as the primary accession numbers to map the annotations information from ClinVar to iPCD. The ClinVar ID, disease, HGVS expression, variant type, annotation, cDNA and corresponding protein information were extracted and integrated into iPCD. At last, 196,913 annotation entries were obtained from the ClinVar.

8.2) GWASdb (http://jjwanglab.org/gwasdb) [53]

First, the 'gwasdb_20150819_snp_drug.gz' file was downloaded from FTP site of GWASdb (ftp://147.8.193.36/GWASdb/gwasdb_20150819_snp_drug.gz, in Oct, 2020). Then, the human gene names (the column entitled 'GENE_SYMBOL') were used to map the corresponding information from GWASdb to EPSD. The annotation columns included 'RefSeq Nucleotide', 'Chr', 'Position', 'SNP ID', 'Ref', 'Alt', 'p-value', 'Drug name', and 'DO Term'. After integrated, we obtained 3255 entries from the GWASdb.

8.3) SNPdbe (https://rostlab.org/services/snpdbe/) [54]

In this work, the compressed file 'SNPdbe_2012_03_05_sql' were downloaded from the 'Download' page of SNPdbe (in Sep, 2020). The compressed file contained 'seqs_refseq.xml', 'seqs_sp.xml', 'seqs_pmd.xml' and 'seqs_containingsnps.xml' four files. First, we extracted the UniProt IDs of proteins from the 'seqs_pmd.xml' file. Next, the positions of SAASs were acquired from the 'seqs_sp.xml' file, and then the associated functional impacts and diseases were extracted from the 'geno2func.xml' file. At last, the UniProt IDs were used to map corresponding annotations of SNPdbe to iPCD. The annotation columns included 'UniProt', 'Position', 'WT', 'Mutation', 'Function' and 'Disease'. After integrated, 46,128 annotation entries were acquired from the SNPdbe.

8.4) ActiveDriverDB (https://activedriverdb.org/) [55]

First, we downloaded two files from the 'Download' page of ActiveDriver, including

‘clinvar_mutations_affecting_ptm_sites.tsv’ and ‘mc3_mutations_affecting_ptm_sites.tsv’ (https://activedriverdb.org/download/, in May, 2021). Then, the Gene names were used to as keys to map corresponding annotation data from ActiveDriverDB to iPCD. These annotation information columns included ‘RefSeq Nucleotide’, ‘Link’, ‘Position’, ‘Mutation alt’, ‘Mutation summary’, ‘Site position’ and ‘Site residue’. After integrated, we obtained 390,262 annotation entries from the ActiveDriverDB.

8.5) BioMuta (https://hive.biochemistry.gwu.edu/biomuta) [56]

In this work, the ‘biomuta-master.csv’ and ‘biomuta-ac2genename.csv’ two files were downloaded from website (https://hive.biochemistry.gwu.edu/beta/biomuta/content/BioMuta3.csv, in Sep, 2020) of BioMuta. Similarly, we used UniProt IDs to map the corresponding data from BioMuta to iPCD. The columns included ‘Ensemble protein’, ‘Position’, ‘Ref (AA)’, ‘Var (AA)’, ‘Chr’, ‘Position (Nuc)’, ‘Ref (Nuc)’, ‘DO name’ and corresponding ‘PMIDs’ were integrated into the annotation file of iPCD. in the end, we obtained 822,811 entries from the BioMuta.

8.6) Kin-Driver (http://kin-driver.leloir.org.ar/) [57]

In this study, we downloaded the ‘kindriver_v82.sql’ file from the ‘Download’ page of Kin-Driver (in May, 2021). In the sql file, we first extracted ‘disease’ and ‘mutant’ two information files. The UniProt IDs were used as the keys to extract necessary annotations from the Kin-Driver by combining disease and mutant two files. The final annotations columns of iPCD include ‘UniProt Name’, ‘Mutation’, ‘Disease’, ‘Rel freq’ and ‘Mutation type’. After integrated, the 733 entries were taken from the Kin-Driver.

8.7) OMIM (http://omim.org/) [58]

The ‘mim2gene.txt’ file was downloaded from the OMIM (in May, 2021). In this work, we used Ensembl gene IDs to map human genes from OMIM to iPCD. For each entry, the annotation column included ‘MIM number’, ‘MIM entry type’, ‘Ensembl gene ID’ and corresponding link were conserved and further to integrated. After the annotation file of iPCD was generated, the entry was counted. Finally, we taken 3238 human genes and corresponding annotations from OMIM.

8.8) PTMD (http://ptmd.biocuckoo.org/) [59]

In 2018, we developed a database PTMD, which contained 1950 known PDAs (PTM-disease associations) in 749 proteins for 23 types of PTMs (Various posttranslational modifications) and 275 types of diseases. In this study, the compressed file 'PTM-Disease association.zip' was downloaded from the 'DOWNLOAD' page of PTMD (in Oct, 2020). The UniProt IDs were used as the keys to map annotation data from PTMD to iPCD. The annotation columns included 'Uniprot', 'Disease', 'PTM', 'Position' 'Type', 'AA' and 'PMIDs' were integrated. After the annotation file of iPCD was generated, the entry was counted. Finally, we taken 1544 PDAs from PTMD.

8.9) MSDD (http://www.bio-bigdata.com/msdd/) [60]

The 'msdd.txt' file were downloaded from download website of MSDD (http://www.bio-bigdata.com/msdd/download.jsp, in Oct, 2020). In this study, the gene names were used as keys to acquired annotation information from MSDD. These columns included 'MSDD ID', 'miRNA', 'Disease', 'SNP position', 'Allele', 'Ancestral Allele', 'Dysfunction Pattern', 'Population', 'Sample Size', 'Case-MAF', 'Control-MAF' and 'PMID' were integrated into iPCD. Finally, we obtained 72 annotated human genes.

8.10) DisGeNET (http://www.disgenet.org) [61]

DisGeNET is database platform, which integrating information on human disease-associated genes and variants. We downloaded the 'all_gene_disease_association.tsv' file from the download page of DisGeNET (in Apr, 2020). We extracted these columns included 'NCBI Gene ID', 'Disease', 'Disease Type', 'Disease Semantic Type', 'Score', and 'EI' from DisGeNET, and then integrated them into iPCD. After the annotation file of iPCD was generated, the entry was counted. Finally, 494,335 entries were taken from DisGeNET.

8.11) DiseaseEnhancer (http://biocc.hrbmu.edu.cn/DiseaseEnhancer/) [62]

We downloaded the two files 'enhInfo-1.0.2.txt' and 'enh2disease-1.0.2.txt' from the website of DiseaseEnhancer (http://biocc.hrbmu.edu.cn/DiseaseEnhancer/JumpToDownload, in Oct, 2020). In this study, we used Gene symbols to map annotation data of DiseaseEnhancer to iPCD. After integrated, the annotation columns included 'Enhancer ID', 'Variant type', 'Variation name',

'Chr', 'Start', 'End', 'Variation mode', 'Disease type' and 'PMIDs'. In the end, we obtained 1023 entries about human genes annotated with disease-associated enhancers.

9. Protein-protein Interaction

9.1) IID (http://iid.ophid.utoronto.ca/) [63]

We downloaded six PPI files from the 'Download' page of IID (*http://iid.ophid.utoronto.ca/*, in Sep, 2020), including 'human_annotated_PPIs.txt', 'mouse_annotated_PPIs.txt', 'rat_annotated_PPIs.txt', 'worm_annotated_PPIs', 'fly_annotated_PPIs.txt' and 'yeast_annotated_PPIs.txt'. In this work, we used UniProt IDs as the keys to map the annotation data from IID to iPCD. The annotation columns included 'Symbol', 'Methods', 'Evidence Type' and 'PMIDs'. After the annotation file of iPCD was generated, the entry was counted. Finally, we obtained 1,309,829 entries in 5 model species from IID.

9.2) BioGRID (https://thebiogrid.org) [64]

BioGRID is a mainstream database of protein-protein interaction. We downloaded 8 .txt files from the download page of BioGRID (https://downloads.thebiogrid.org/BioGRID/Release-Archive/BIOGRID-4.0.189/, in May, 2021), including 'BIOGRID-ORGANISM-Arabidopsis_thaliana_Columbia-4.4.197.tab3', 'BIOGRID-ORGANISM-Caenorhabditis_elegans-4.4.197.tab3', 'BIOGRID-ORGANISM-Danio_rerio-4.4.197.tab3', 'BIOGRID-ORGANISM-Drosophila_melanogaster-4.4.197.tab3', 'BIOGRID-ORGANISM-Homo_sapiens-4.4.197.tab3', 'BIOGRID-ORGANISM-Mus_musculus-4.4.197.tab3', 'BIOGRID-ORGANISM-Rattus_norvegicus-4.4.197.tab3' and 'BIOGRID-ORGANISM-Saccharomyces_cerevisiae_S288c-4.4.197.tab3'. These annotation columns included 'UniProt ID', 'Gene name', 'Experimental System' and 'Throughput' were integrated into iPCD by mapping the UniProt ID. Finally, we obtained 936,105 protein-protein interaction entries from the BioGRID.

9.3) iRefIndex (http://irefindex.org) [65]

In this work, we first downloaded the file 'All.mitab.22012018.txt.zip' from iRefIndex website (http://irefindex.org/download/irefindex/data/archive/release_15.0/psi_mitab/MITAB2.6/, in Jun, 2020). Similarly, the UniProt IDs were used to map the information of PPI from iRefIndex to iPCD.

After integrated, the annotation columns included ‘UniProt ID’, ‘Method’, ‘Interaction type’, ‘Sourcedb’, ‘Confidence’ and ‘PMIDs’ were integrated. In the end, 865,915 pairs of PPIs in 8 model organisms were obtained from the iRefIndex.

9.4) PINA (http://cbg.garvan.unsw.edu.au/pina) [66]

In this study, 7 PPI files for 7 model species were downloaded from the download website of PINA (http://omics.bjcancer.org/pina/interactome.stat.do, in Oct, 2020), including ‘Arabidopsis thaliana-20140521.tsv’, ‘Caenorhabditis elegans-20140521.tsv’, ‘Drosophila melanogaster-20140521.tsv’, ‘Homo sapiens-20140521.tsv’, ‘Mus musculus-20140521.tsv’, ‘Rattus norvegicus-20140521.tsv’ and ‘Saccharomyces cerevisiae-20140521.tsv’. First, the UniProt IDs were used to map the PPI pairs of PINA to iPCD. Then, these columns entitled ‘Uniprot ID’, ‘Gene name’, ‘Methods’, ‘Interaction types’, ‘Source databases’, and ‘PMIDs’ were integrated. After the annotation file of iPCD was generated, the entry was counted. Finally, we acquired 224,980 entries of PPIs from the PINA.

9.5) HINT (http://hint.yulab.org) [67]

In this study, 7 PPI files for 7 model organisms were downloaded from HINT website (http://hint.yulab.org/download/, in Oct, 2020), including ‘HomoSapiens_cocomp_hq.txt’, ‘SaccharomycesCerevisiaeS288C_cocomp_hq.txt’, ‘ArabidopsisThaliana_cocomp_hq.txt’, ‘DrosophilaMelanogaster_cocomp_hq.txt’, ‘CaenorhabditisElegans_cocomp_hq.txt’, ‘RattusNorvegicus_cocomp_hq.txt’ and ‘MusMusculus_cocomp_hq.txt’. The UniProt IDs were adopted to map PPI pairs of HINT to iPCD. We retained the information in columns including ‘Uniprot’, ‘Uniprot ID’, ‘Gene name’, ‘Method’, ‘Quality’ and ‘PMIDs’. Finally, we obtained 136,648 pairs of PPIs from HINT.

9.6) Mentha (http://mentha.uniroma2.it) [68]

In this work, the file ‘all.zip’ were downloaded from the download page of Mentha (http://mentha.uniroma2.it/download.php, in Oct, 2020). First, the UniProt IDs were adopted as primary accession numbers to map the corresponding PPI data from Mentha to iPCD. After integrated, the annotation columns included ‘Score’ and ‘PMIDs’. Here, we obtained 388,293 pairs

of PPIs from Mentha.

9.7) inBio Map^{TM} (http://www.intomics.com/inbio/map) [69]

First, we downloaded the compressed file 'InBio_Map_core_2016_09_12.tar.gz' from download page of inBio Map^{TM} (https://www.intomics.com/inbio/map.html#downloads, in Sep, 2020). Then, the UniProt IDs were adopted as primary accession numbers to map the corresponding PPIs data from inBio Map^{TM} to iPCD. After integrated, the annotation columns of iPCD included 'UniProt', 'UniProt ID', 'Gene name', 'Method' and 'Source'. Finally, 412,533 pairs of PPIs were obtained in human.

9.8) STRING (https://string-db.org/) [70]

We downloaded the compressed file 'protein.links.detailed.v11.0.txt.gz' of 8 model species from the 'Download' page of STRING (in Sep, 2020). In this work, the Ensembl protein IDs were used as the keys to map PPI pairs from STRING to iPCD. After integrated, the annotation columns of iPCD included 'Ensembl protein', 'Ensembl protein ID', 'Neighborhood', 'Gene fusion', 'Coocurence', 'Coexpression', 'Experimental', 'Database', 'Textmining' and 'Score'. After the annotation file of iPCD was generated, the entry was counted. Finally, 22,614,986 pairs of PPIs were obtained from STRING in 8 model organisms, including *H. sapiens, M. musculus, R. norvegicus, D. melanogaster, C. elegans, S. cerevisiae, A. thaliana, and D. rerio*.

9.9) TIMBAL (http://mordred.bioc.cam.ac.uk/timbal) [71]

First, we downloaded the 'TIMBAL_sm.csv' file from download website of TIMBAL (http://mordred.bioc.cam.ac.uk /timbal/all, in May, 2021). Then, we used the UniProt IDs to map PPI pairs from TIMBAL to iPCD. the 'Literature name', 'PDB code', 'Activity in paper', 'Activity comment', 'Activity context', 'Assay description', 'Assay type', 'Confidence description', and 'PMIDs' were conserved and integrated. After the annotation file of iPCD was generated, the entry was counted. Finally, we obtained 16,550 pairs of PPIs from TIMBAL.

10. Drug-target relation

10.1) TTD (http://bidd.nus.edu.sg/group/cjttd/) [72]

The ‘P1-01-TTD_download.txt’ file were downloaded from the download page of TTD website (https://db.idrblab.org/ttd/full-data-download, in Nov, 2020). First, we used the UniProt IDs as primary accession numbers to find drug targeting proteins in iPCD. For each entry, the Drug ID, Drug names, Target validation and corresponding links were extracted and conserved. After the annotation file of iPCD was generated, the entry was counted. Finally, 24,221 records of drug-target relations were acquired from the TTD.

10.2) DrugBank (https://www.drugbank.ca/) [73]

In this study, the compressed file ‘drugbank_all_full_database.xml.zip’ was downloaded from the download page of DrugBank (https://www.drugbank.ca/releases/latest, in Nov, 2020). First, the UniProt IDs were used as primary accession numbers to map corresponding data from DrugBank to iPCD. For each entry, the DrugBank IDs, drug names, groups, know-action and corresponding links were extracted and conserved. After the annotation file of iPCD was generated, the entry was counted. Finally, 6718 records of drug-target relations were acquired from the DrugBank.

10.3) GtoPdb (http://www.guidetopharmacology.org/targets.jsp) [74]

First, the ‘interactions.csv’ file were downloaded from the download website of GtoPdb (http://www.guidetopharmacology.org/download.jsp, in Oct, 2020). And then, the UniProt IDs were used as primary accession numbers to find drug targeting proteins in iPCD. After integrated, the annotation columns included ‘Target ID’, ‘Ligand’, ‘Type’, ‘PMIDs’ and corresponding link for each entry. After the annotation file of iPCD was generated, the entry was counted. Finally, 8664 records of drug-target relations were acquired from the GtoPdb.

10.4) ADReCS-Target (http://bioinf.xmu.edu.cn/ADReCS-Target) [75]

In this work, we downloaded the file ‘P_D_A.xlsx’ from the download page of ADReCS-Target (http://bioinf.xmu.edu.cn/ADReCS-Target/download.jsp, in Oct, 2020). Similarly, the UniProt IDs were used as keys to find drug targeting proteins in iPCD. After integrated, the annotation columns included ‘UniProt’, ‘Interaction’, ‘Drug name’ and ‘ADR Term’. After the annotation file of iPCD was generated, the entry was counted. Finally, 1228 records of drug-target relations were acquired from the ADReCS-Target.

10.5) ECOdrug (http://www.ecodrug.org) [76]

First, from the download page of ECOdrug (http://www.ecodrug.org/#downloads, in May, 2021), we downloaded the file 'ECOdrug_ensembl.csv'. Next, the Ensembl gene IDs as primary accessions to map the corresponding annotation data from ECOdrug to iPCD. And then, the 'Interaction', 'Drug_name', 'Drug_type', and 'Drug_first approval' were conserved and integrated for each entry. After the annotation file of iPCD was generated, the entry was counted. Finally, 6,968 records of drug-target relations were acquired from the ECOdrug.

10.6) DGIdb (http://www.dgidb.org/) [77]

In this study, we downloaded the file 'interactions.tsv' from the download page of DGIdb (http://www.dgidb.org/downloads, in Sep, 2020). First, the NCBI Gene IDs as primary accession numbers to map drug-target relations from DGIdb to iPCD. After integrated, the annotation columns included 'Drug claim name', 'Drug claim primary name', 'Drug name' and corresponding link for each entry. At last, we obtained 22,452 entries from the DGIdb about drug-target.

10.7) CTD (http://ctdbase.org/) [78]

In this study, from the download page of CTD (http://ctdbase.org/downloads/, in May, 2021), we downloaded the file 'CTD_chem_gene_ixns.tsv.gz'. First, we used the NCBI Gene IDs as primary accessions to map drug-target relations from CTD to iPCD. And then, the 'Chemical name', 'Interaction types', 'Interaction actions' and 'PMIDs' were conserved and integrated. After the annotation file of iPCD was generated, the entry was counted. Finally, 935,687 records of drug-target relations were acquired from the CTD.

10.8) DrugCentral (http://drugcentral.org/) [79]

The compressed file 'drug.target.interaction.tsv' was downloaded from the download page of DrugCentral (http://drugcentral.org/download, in May, 2020). Similarly, the UniProt IDs were used as the indexes to map corresponding data from DrugCentral to iPCD. The columns included 'Drug name', 'Action type' and corresponding link were conserved and integrated into iPCD. After the annotation file of iPCD was generated, the entry was counted. Finally, 6890 records of drug-target

relations were acquired from the DrugCentral.

11. Orthologous information

11.1) InParanoid (http://inparanoid.sbc.su.se/cgi-bin/index.cgi) [80]

First, we downloaded the compressed file 'InParanoidUniProtXref' from the 'Downloads' page of InParanoid (in May 2021). And then, the UniProt IDs were extracted as primary accession numbers to map the orthologs inferred data of InParanoid to iPCD for the 8 model organisms. After the annotation file of iPCD was generated, the entry was counted. Finally, we obtained 15,941 entries from the InParanoid.

11.2) OMA (https://omabrowser.org/oma/) [81]

In this study, we downloaded the compressed file 'oma-uniprot.txt' from the 'Download' page of OMA (in May 2021). Then, we used the UniProt IDs as primary accessions to map the orthologs inferred data from OMA to iPCD for the 8 model organisms. After the annotation file of iPCD was generated, the entry was counted. Finally, we obtained 24,335 entries from the OMA.

11.3) OrthoDB (http://www.orthodb.org/) [82]

We downloaded two compressed files 'odb10v0_genes.tab' and 'odb10v0_OG2genes.tab' in the 'Downloads' page of OrthoDB (https://www.orthodb.org/?page=filelist, in May 2021). First, we extracted the UniProt IDs and OrthoDB gene IDs from the file 'odb10v0_genes.tab', and then OrthoDB IDs were retrieved from the file 'odb10v0_OG2genes.tab'. At last, the UniProt IDs were adopted as primary accession numbers to map the orthologs inferred data from OrthoDB to iPCD for 8 model species. After the annotation file of iPCD was generated, the entry was counted. Finally, we obtained 89,085 entries from the OrthoDB.

12. Biological pathway

12.1) KEGG (http://www.genome.jp/kegg/) [83]

We downloaded three files 'pathway.list', 'genes_uniprot.list' and 'gene_map.tab' for 8 model species from the FTP server of KEGG (Kyoto Encyclopedia of Genes and Genomes) (ftp.bioinformatics.jp/kegg, in Jun, 2021). First, the UniProt IDs were adopted as primary accession

number to map the pathway data of KEGG to iPCD. And then, the KEGG ID, pathway ID, and corresponding description and link were integrated into the annotation file of iPCD. Finally, 49,438 biological pathways information were obtained from the KEGG.

12.2) SignaLink (http://signalink.org/) [84]

In this work, the file '05032019-signalink-TuI8g3.csv' was downloaded from the 'download' page of SignaLink (in Oct, 2020). We used the UniProt IDs as primary accessions to map the corresponding pathway data of SignaLink to iPCD. The annotation information columns included 'Source name', 'Target name', 'Target UniProt ID', 'Interaction type', 'Directness', 'PMIDs' and corresponding link for each entry were integrated. Finally, 35,721 biological pathways information were obtained from the SignaLink.

12.3) PathBank (http://www.pathbank.org) [85]

We downloaded two files 'pathbank_all_pathways.csv.zip' and 'pathbank_all_proteins.csv.zip' from the PathBank (in Nov, 2020). First, the UniProt IDs were use as the keys to map pathway data of PathBank to iPCD. After integrated, the annotation columns included 'PathBank ID', 'Category' and corresponding description and link for each entry. Finally, 119,357 biological pathways information were obtained from the PathBank.

12.4) Reactome (https://reactome.org/) [86]

We acquired the file 'UniProt2Reactome.txt' from the 'Download' page of Reactome (Version 68, in Dec 2020). In this study, we used the UniProt IDs as primary accessions to map the pathway data of Reactome to iPCD for the 8 model organisms. For each entry, the Reactome ID and corresponding description were extracted and integrated. Finally, 47,486 biological pathways information were obtained from the Reactome.

13. Transcriptional regulator

13.1) TRRUST (http://www.grnpedia.org/trrust/) [87]

We downloaded the two files 'trrust_rawdata.human.tsv' and 'trrust_rawdata.mouse.tsv' from download page of TRRUST (http://www.grnpedia.org /trrust/downloadnetwork.php, in Jun, 2021).

First, we adopted Gene names as primary keys to map transcriptional regulator data from TRRUST to iPCD. After integrated, the annotation columns included 'Species,Name', 'TF', 'Mode of Regulation' and 'PMIDs'. Finally, 10,197 proteins annotated with transcriptional regulators were obtained from the TRRUST.

13.2) HEDD (http://zdzlab.einstein.yu.edu/1/hedd/hedd.php) [88]

In this work, we downloaded the two files 'DataDownload_Enhancer.txt' and 'DataDownload_EnhancerTagretGene.txt' from the 'Download Data' page of HEDD (http://zdzlab.einstein.yu.edu/1/hedd/download.php, in May, 2021). We adopted the Gene names to map transcriptional regulator data from HEDD in the file 'DataDownload_EnhancerTagretGene.txt'. In addition, we acquired the information of enhancers in the file 'DataDownload_Enhancer.txt'. Finally, 151,073 proteins annotated with transcriptional regulators were obtained from the HEDD.

13.3) DroID (http://droidb.org/) [89]

First, the file 'tf_gene.txt' was downloaded from the 'Downloads' page of DroID (http://droidb.org/Downloads.jsp, in May, 2021). The Gene names in column 'GENE_SYMBOL' were adopted as the primary keys to map transcriptional regulator data from DroID to iPCD. After integrated, the annotation columns included 'TF ID', 'TF', 'Method' and 'PMIDs'. Finally, 26,223 proteins annotated with transcriptional regulators were obtained from the DroID.

13.4) YTRP (http://cosbi3.ee.ncku.edu.tw/YTRP/) [90]

In this work, the 'TRP_direct_regulatory_network.txt' was downloaded from the download page of YTRP (http://cosbi3.ee.ncku.edu.tw/YTRP/Download, in May, 2021). And then, the gene symbols as primary keys to map transcriptional regulator data from YTRP to iPCD. The columns entitled 'TF' and 'Experimental Condition' were conserved and integrated. Finally, 2314 proteins annotated with transcriptional regulators were obtained from the YTRP.

13.5) RegNetwork (http://www.regnetworkweb.org/) [91]

The ‘human.zip’ and ‘mouse.zip’ two files were downloaded from the download page of RegNetwork (http://www.regnetworkweb.org/download.jsp, in May, 2021). And then, the Gene symbols in column ‘TARGET SYMBOL’ was adopted as primary keys to map corresponding data from RegNetwork to iPCD. After integrated, the annotation columns included ‘NCBI Gene ID’, ‘Regulator ID’ and ‘Regulator’. Finally, 159,104 proteins annotated with transcriptional regulators were obtained from the RegNetwork.

13.6) TCGA (https://www.cancer.gov/tcga) [12]

The ‘gdac.broadinstitute.org_ACC.Methylation_Preprocess.Level_3.2016012800.0.0.tar.gz’ file was download from the BROAD Institute (in Jun, 2021) for 37 cancers. In this work, the gene name in the column entitled ‘Gene_Symbol’ was adopted as the keys to map methylation information from the TCGA to iPCD. After integrated, the annotations columns included ‘Gene-Symbol’, ‘Chromosome’, ‘Genomic_Coordinate’, ‘Cancer type’, ‘Sample’ and “Beta_value’. Finally, 3186 PCD proteins were annotated with methylation of TCGA.

14. mRNA expression

14.1) TCGA (https://www.cancer.gov/tcga) [12]

All file about mRNA expression of TCGA for 37 cancers were downloaded from BROAD Institute (http://gdac.broadinstitute.org/runs/stddata__2016_01_28/data, Oncotated calls, level 3, in Jun, 2021). In this work, we acquired the mRNA expression level of each patient sample from the ‘ACC.uncv2.mRNAseq_RSEM_normalized_log2_PARADIGM.txt’, and then, the Entrez IDs were adopted as the keys to map mRNA expression data from TCGA to iPCD. After integrated, the annotation columns included ‘HYBRIDIZATION R’, ‘Cancer type’, ‘Sample’ and ‘Expression’. At last, we obtained 48,679,186 entries for 37 cancers from TCGA.

14.2) ICGC (http://icgc.org/) [13]

First, all available gene expression profiles for 37 ICGC projects were download by using sequencing-based platforms, including BLCA-US, BOCA-FR, BPLL-FR, BRCA-KR, BRCA-US, CESC-US, CLLE-ES, COAD-US, GBM-US, HNSC-US, KIRC-US, KIRP-US, LAML-US, LGG-US, LICA-FR, LIHC-US, LIRI-JP, LUAD-US, LUSC-US, MALY-DE, ORCA-IN, OV-AU, OV-US, PAAD-

US, PACA-AU, PACA-CA, PAEN-AU, PBCA-US, PRAD-CA, PRAD-FR, PRAD-US, READ-US, RECA-EU, SKCM-US, STAD-US, THCA-US and UCEC-US, from the ICGC data portal (https://dcc.icgc.org/releases/release_28/Projects/, in Oct, 2020). In this work, the Ensembl gene IDs were adopted to map mRNA expression data from ICGC to iPCD. After integrated, we obtained 36,534,103 mRNA expression entries from ICGC.

14.3) ArrayExpress (https://www.ebi.ac.uk/arrayexpress) [92]

In this study, we downloaded six files from the FTP site of ArrayExpress (ftp://ftp.ebi.ac.uk/pub/databases/arrayexpress/data/, in Dec 2020), including 'allgenes_nonde_in_normal_2.0.14.tab', 'allgenes_nonde_in_organism_part_2.0.14.tab,' 'allgenes_updown_in_disease_2.0.19.tab', 'allgenes_updown_in_normal_2.0.14.tab', 'allgenes_updown_in_organism_part_2.0.14.tab' and 'organism_part_atlas_13.07.tab'. The Ensembl gene IDs were adopted to map mRNA expression data from ArrayExpress to iPCD. The columns entitled 'Experimental Factor', 'Factor Value', 'Experiment Accession', 'Expression' and 'p Value' were conserved and integrated. Finally, we obtained 5,108,126 mRNA expression entries from the ArrayExpress.

14.4) GXD (http://www.informatics.jax.org/expression.shtml) [93]

The two files 'MRK_GXDAssay.rpt.txt' and 'MGI_Gene_Model_Coord.rpt.txt' were downloaded from its website (http://www.informatics.jax.org/mgihome/GXD/aboutGXD.shtml, in Jun, 2021). We adopted the Ensembl gene IDs to map mRNA expression data from GXD to iPCD. For each entry, the 'Assay', 'Marker name' and corresponding link were extracted and integrated. Finally, we obtained 37,928 mRNA expression entries from the GXD.

14.5) COSMIC (https://cancer.sanger.ac.uk/cosmic) [14]

In this work, we downloaded the compressed file 'CosmicCompleteGeneExpression.tsv' from the 'Downloads' page of COSMIC (https://cancer.sanger.ac.uk/cosmic/download, in Jul, 2020). The gene symbols were used to map mRNA expression data from COSMIC to iPCD. After integrated, the annotation information included gene name, sample ID, regulation, and Z-score. Finally, we obtained 30,557,962 mRNA expression entries from COSMIC.

14.6) BioXpress (https://hive.biochemistry.gwu.edu/bioxpress) [56]

First, the file 'BioXpress_gene_differential_expression_v2.0.csv' were downloaded from its website (https://hive.biochemistry.gwu.edu/bioxpress, in Dec 2020). And then, the UniProt IDs were used as the keys to map corresponding mRNA expression data from BioXpress to iPCD. The columns entitled 'log_2(FoldChange)', 'p-value', 'Significant', 'Trend', 'TCGA Cancer', 'Cancer Ontology', 'Patients' and 'UBERON_ID' were extracted and integrated. Finally, we obtained 3415 mRNA expression entries from the BioXpress.

14.7) TissGDB (http://zhaobioinfo.org/TissGDB) [94]

In this study, we downloaded two files 'Tissg_DEGs' and 'TissgDB_basic_uniq.txt' from the 'Download' page of TissGDB (https://bioinfo.uth.edu/TissGDB/download.html, in Nov, 2020). The columns entitled 'Cancer Type', 'Tumor', 'Normal', 'log2(FC)', 'p-value' and 'FDR' were extracted from 'Tissg_DEGs'. The UniProt IDs and gene symbols were extracted from 'TissgDB_basic_uniq.txt'. Similarly, we used UniProt IDs as the keys to map corresponding mRNA expression data from TissGDB. Finally, we obtained 1,325 mRNA expression entries.

14.8) FFGED (http://bioinfo.townsend.yale.edu/) [95]

We downloaded all 13,420 XML profiles from the FTP server of FFGED. In this work, we adopted the Ensembl gene IDs as the primary accessions to map mRNA expression data from FFGED to iPCD. After integrated, the annotation columns included 'Experiment ID', 'Experiment Name', 'Expression Variable', 'Expression Value' and corresponding link for each entry. Finally, we obtained 22,372 entries from the FFGED.

14.9) TISSUES (http://tissues.jensenlab.org/) [96]

First, we downloaded all knowledge, experiments and text mining files of H. sapiens, M. musculus and R. norvegicus from the 'Downloads' page of TISSUES (https://tissues.jensenlab.org/Downloads, in Nov, 2020), including 'human_tissue_experiments_full.tsv', 'human_tissue_knowledge_full.tsv', 'human_tissue_textmining_full.tsv', 'mouse_tissue_experiments_full.tsv',

‘mouse_tissue_knowledge_full.tsv’, and ‘mouse_tissue_textmining_full.tsv’. We used Ensembl protein IDs and gene names to map corresponding data from the TISSUES to iPCD. After integrated, the annotation columns included ‘Ensembl protein ID’, ‘Tissue ID’, ‘Tissue’, ‘Source’, ‘Evidence/ Z-score’, and ‘Confidence’. Finally, we obtained 6,478,895 mRNA expression entries from the TISSUES.

15. Protein expression/Proteomics

15.1) The Human Protein Atlas (HPA) (http://www.proteinatlas.org/) [97]

First, we acquired the compressed file ‘proteinatlas.xml’ from the ‘DOWNLOADABLE DATA’ page of HPA (https://www.proteinatlas.org/about/download, in Nov, 2020). Here, the UniProt IDs were used as the keys to map corresponding data from HPA to iPCD. The ‘Tissue’ and ‘Score’ were extracted and integrated. Finally, 95,924 tissue protein expression entries were obtained from the HPA.

15.2) Human Proteome Map (HPM) (http://www.humanproteomemap.org/) [98]

We downloaded the file ‘HPM_protein_level_expression_matrix_Kim_et_al_052914.csv’ from the ‘Download’ page of HPM (http://www.humanproteomemap.org/download_hpm_data.php, in Nov, 2020). In this work, the RefSeq protein IDs in the column ‘RefSeq Accession’ were adopted as keys to map corresponding protein expression data from HPM to iPCD. We integrated the protein expression of 17 adult tissues, 6 primary hematopoietic cells and 7 fetal tissues. And the annotation columns included ‘Tissue/Cell line’ and ‘Expression’. Finally, 113,392 tissue protein expression entries were obtained from the HPM.

16. DNA & RNA Element

16.1) circBase (http://www.circbase.org/) [99]

We acquired the ‘hsa_hg19_circRNA.txt’ file from the download website of circBase (http://www.circbase.org/cgi-bin/downloads.cgi, in Nov 2020). In this work, the Refseq IDs were adopted as the primary keys to map cirRNAs information form the circBase to iPCD. The columns entitled ‘RefSeq Nucleotide’, ‘circBase ID’, ‘Chromosome’, ‘Start position’, ‘End position’, ‘Strand’

and ‘Sample’ were conserved and integrated. Finally, 24,264 circRNAs entries were taken from the circBase.

16.2) TransCirc (https://www.biosino.org/transcirc/) [100]

First, the ‘transcirc_metadata.tsv’ file was downloaded from the TransCirc (in Jun, 2021). Then, we used the Ensembl gene ID in the columns entitled ‘gene_id’ as the primary accession numbers to map corresponding data from the TransCirc to iPCD. The ‘TransCirc ID’, ‘Xref’, and corresponding link were conserved and integrated. After the annotation file of iPCD was generated, the entry was counted. At last, we obtained 71,199 entries from the TransCirc.

16.3) TargetScan (http://www.targetscan.org/vert_72/) [101]

We downloaded three files ‘Fly-Conserved_Site_Context_Scores.txt.zip’, ‘Human-Conserved_Site_Context_Scores.txt.zip’ and ‘Mouse-Conserved_Site_Context_Scores.txt.zip’ from the TargetScan (in Jun, 2021). The Ensembl transcript IDs were adopted as the primary keys to acquire necessary information from the TargetScan. After integrated, the annotation columns included ‘Ensembl transcript’, ‘miRNA’, ‘UTR start’, ‘UTR end’, ‘Context++ score’, ‘Context++ score percentile’, ‘Weighted context++ score’ and ‘Weighted context++ score percentile’. After the annotation file of iPCD was generated, the entry was counted. Finally, we obtained 481,526 entries from the TargetScan.

16.4) miRWalk (hhttp://mirwalk.umm.uni-heidelberg.de/) [102]

In this work, we acquired all file from the downloaded website of miRWalk (http://mirwalk.umm.uniheidelberg.de/resources/, in Jun, 2021), including ‘hsa_miRWalk_3UTR.zip’, ‘hsa_miRWalk_5UTR.zip’, ‘mmu_miRWalk_3UTR.zip’ and ‘mmu_miRWalk_5UTR.zip’. The RefSeq IDs were adopted as the keys to map corresponding data from the miRWalk to iPCD. The annotation columns included ‘miRNA’, ‘mRNA’, ‘Genesymbol’, ‘binding_site’ and ‘binding_probability’ were extracted and integrated. Finally, we obtained 29,827,428 entries from the miRWalk.

16.5) miRcode (http://mircode.org/) [103]

First, we downloaded ‘mircode_highconsfamilies.txt.gz’ from the download website of miRcode (http://mircode.org/download.php/, in Jun, 2021). And then, the Ensembl gene IDs in the columns entitled ‘gene_id’ were adopted to map corresponding data from the miRcode to iPCD. The ‘miRNA’, ‘Position’, ‘Transcript region’, ‘Seed pos’, ‘Seed type total conservation (%)’, ‘Primates conservation (%)’, ‘Mammals conservation (%)’ and ‘Vertebrates conservation (%)’ were extracted. after integrated, we acquired 195,642 entries from the miRcode.

16.6) RNAInter (http://www.rna-society.org/rnainter/) [104]

From the RNAInter (http://www.rna-society.org/rnainter/download.html, in Oct, 2020), the file ‘RNA-Protein.zip’ were taken. We used gene IDs as the keys to acquired corresponding data from the RNAInter. The ‘RNAIter ID’, ‘Category’, ‘Symbol’ and ‘Confidence Score’ were conserved and integrated. Finally, we acquired 9,436,583 entries from the RNAIter.

16.7) SEA (http://sea.edbc.org) [105]

All available files were downloaded from the SEA for 5 model organisms, including *H. sapiens* (SEA00101.bed), *M. musculus* (SEA00201.bed), *C. elegans* (SEA00301.bed), *D. melanogaster* (SEA00401.bed) and *D. rerio* (SEA00501.bed). The gene names were used as the primary accessions to map data from the SEA to iPCD. The annotation columns included ‘SEA ID’, ‘SE name’, ‘Chromosome’, ‘Start position’, ‘End position’, ‘Cell/Tissue type’ and ‘Factor’ were integrated. Finally, we obtained 460,893 entries from the SEA.

16.8) miRNAMap (http://mirnamap.mbc.nctu.edu.tw/) [106]

All available files were acquired from the miRNAMap for 5 model organisms, including *H. sapiens* (miRNA_targets_hsa.txt), *M. musculus* (miRNA_targets_mmu), *C. elegans* (miRNA_targets_cel.txt), *R. norvegicus* (miRNA_targets_rno.txt) and *D. rerio* (miRNA_targets_dre.txt). in this work, the Ensembl transcript ID were used as the primary accession numbers to map miRNA targets information from the miRNAMap to iPCD. The columns ‘mature miRNA’, ‘target start’, ‘target end’, miRNA 3-5’, ‘alinment’, ‘target 5-3’, and ‘tool name’ were extracted and integrated. Finally, we obtained 386,452 entries from the miRNAMap.

16.9) miRecords (http://c1.accurascience.com/miRecords/) [107]

From the miRecords (http://c1.accurascience.com/miRecords/download.php, in Nov, 2020), we acquired the file 'miRecords_version4.xls' from its download page. In this study, the RefSeq IDs were used as the indexes to match miRNA-target information from the miRecords to iPCD. The miRNA, target site and corresponding PMIDs were extracted and conserved. After integrated, 1541 miRNA-target interactions were obtained.

16.10) miRTarBase (http://miRTarBase.mbc.nctu.edu.tw/) [108]

From the miRTarBase, the 'miRTarBase_MTI.xlsx' were obtained from its download website (https://mirtarbase.cuhk.edu.cn/~miRTarBase/miRTarBase_2019/php/download.php, in Nov, 2020). The Gene ID were adopted as the keys to acquire the miRNA-target information from the miRTarBase. The 'miRTarBase ID', 'miRNA', 'Experiments', 'Support Type' and 'PMIDs' were extracted and integrated into iPCD.

17. Subcellular localization

17.1) NLSdb (https://rostlab.org/services/nlsdb/) [109]

From the NLSdb (https://rostlab.org/services/nlsdb/downloads, in Oct, 2021), the 'signals.csv' and 'extsignals.csv' files were downloaded from its 'Downloads' page. First, the UniProt IDs in the column 'Origin' were used to map localization information from the NLSdb. And then, the annotation columns included 'Sequence', 'Signal Type', 'Annotation Type', 'Confidence (proteins)' and 'Confidence (families)' were integrated. Finally, we taken 801 subcellular localization entries from the NLSdb.

17.2) COMPARTMENTS (https://compartments.jensenlab.org/) [110]

From the COMPARTMENTS (https://compartments.jensenlab.org/Downloads, in Oct, 2020), all .tsv files in 7 model organisms were obtained, including *H. sapiens* (human_compartment_integrated_full), *M. musculus* (mouse_compartment_integrated_full), *R. norvegicus* (rat_compartment_integrated_full), *D. melanogaster* (fly_compartment_integrated_full), *C. elegans* (worm_compartment_integrated_full), *A. thaliana (arabidopsis_compartment_integrated_full)*, *S. cerevisiae* (yeast_compartment_integrated_full).

The Ensembl protein IDs were adopted to map subcellular localization data to iPCD. The annotation columns 'COMPARTMENTS ID', 'Primary name', 'GO ID', 'Name' and 'Confidence' were extracted and integrated. Finally, 2,717,137 entries were taken from the COMPARTMENTS.

17.3) Membranome (http://membranome.org) [111]

From the Membranome (https://membranome.org/download, in May, 2021), the 'proteins-2020-10-05.csv' file were acquired from its download page. in this work, we adopted UniProt accession numbers as the keys to match subcellular localization data to iPCD. The protein family and localization were extracted and integrated. Finally, 556 protein subcellular localization were obtained from the Membranome.

17.4) Translocatome (http://translocatome.linkgroup.hu) [112]

From the Translocatome (http://translocatome.linkgroup.hu/download, in Oct, 2020), we acquired three files 'translocatome_all_data.csv', 'translocatome_transloc_data.csv' and 'translocatome_nontransloc_data.csv' from its 'DOWNLOAD' page. First, the UniProt IDs were adopted as the primary keys to map corresponding information from the Translocatome to iPCD. And then, the 'Translocation evidence score', 'Translocation evidence score Classification' and corresponding 'PMIDs' and Translocatome links were extracted and integrated. At last, we obtained 3134 entries from the Translocatome.

Supplementary references

1. Díez J, Walter D, Muñoz-Pinedo C et al. DeathBase: a database on structure, evolution and function of proteins involved in apoptosis and other forms of cell death, Cell Death and Differentiation 2010;17:735-736.
2. Wanichthanarak K, Cvijovic M, Molt A et al. yApoptosis: yeast apoptosis database, Database (Oxford) 2013;2013:bat068.
3. Moussay E, Kaoma T, Baginska J et al. The acquisition of resistance to TNFα in breast cancer cells is associated with constitutive activation of autophagy as revealed by a transcriptome analysis using a custom microarray, Autophagy 2011;7:760-770.
4. Homma K, Suzuki K, Sugawara H. The Autophagy Database: an all-inclusive information resource on autophagy that provides nourishment for research, Nucleic Acids Research 2011;39:D986-990.
5. Türei D, Földvári-Nagy L, Fazekas D et al. Autophagy Regulatory Network - a systems-level bioinformatics resource for studying the mechanism and regulation of autophagy, Autophagy 2015;11:155-165.
6. Wang NN, Dong J, Zhang L et al. HAMdb: a database of human autophagy modulators with specific pathway and disease information, J Cheminform 2018;10:34.
7. Chen K, Yang D, Zhao F et al. Autophagy and Tumor Database: ATdb, a novel database connecting autophagy and tumor, Database (Oxford) 2020;2020.
8. Zhou N, Bao J. FerrDb: a manually curated resource for regulators and markers of ferroptosis and ferroptosis-disease associations, Database (Oxford) 2020;2020.
9. Zhang L, Zhang L, Guo Y et al. MCDB: A comprehensive curated mitotic catastrophe database for retrieval, protein sequence alignment, and target prediction, Acta Pharm Sin B 2021;11:3092-3104.
10. Arntzen M, Bull VH, Thiede B. Cell death proteomics database: consolidating proteomics data on cell death, J Proteome Res 2013;12:2206-2213.
11. Wu D, Huang Y, Kang J et al. ncRDeathDB: A comprehensive bioinformatics resource for deciphering network organization of the ncRNA-mediated cell death system, Autophagy 2015;11:1917-1926.
12. Hutter C, Zenklusen JC. The Cancer Genome Atlas: Creating Lasting Value beyond Its Data, Cell 2018;173:283-285.
13. Zhang J, Bajari R, Andric D. The International Cancer Genome Consortium Data Portal, Nature Biotechnology 2019;37:367-369.
14. Tate JG, Bamford S, Jubb HC et al. COSMIC: the Catalogue Of Somatic Mutations In Cancer, Nucleic Acids Research 2019;47:D941-d947.
15. Sherry ST, Ward MH, Kholodov M et al. dbSNP: the NCBI database of genetic variation, Nucleic Acids Research 2001;29:308-311.
16. Gundem G, Perez-Llamas C, Jene-Sanz A et al. IntOGen: integration and data mining of multidimensional oncogenomic data, Nature Methods 2010;7:92-93.
17. Wagih O, Reimand J, Bader GD. MIMP: predicting the impact of mutations on kinase-substrate phosphorylation, Nature Methods 2015;12:531-533.
18. Li J, Shi L, Zhang K et al. VarCards: an integrated genetic and clinical database for coding variants in the human genome, Nucleic Acids Research 2018;46:D1039-d1048.

19. Ning W, Guo Y, Lin S et al. DrLLPS: a data resource of liquid-liquid phase separation in eukaryotes, Nucleic Acids Research 2020;48:D288-d295.
20. Guo Y, Peng D, Zhou J et al. iEKPD 2.0: an update with rich annotations for eukaryotic protein kinases, protein phosphatases and proteins containing phosphoprotein-binding domains, Nucleic Acids Research 2019;47:D344-d350.
21. Zhou J, Xu Y, Lin S et al. iUUCD 2.0: an update with rich annotations for ubiquitin and ubiquitin-like conjugations, Nucleic Acids Research 2018;46:D447-d453.
22. Xu Y, Zhang S, Lin S et al. WERAM: a database of writers, erasers and readers of histone acetylation and methylation in eukaryotes 2017;45:D264-d270.
23. Hu H, Miao YR, Jia LH et al. AnimalTFDB 3.0: a comprehensive resource for annotation and prediction of animal transcription factors, Nucleic Acids Research 2019;47:D33-d38.
24. Jin J, Tian F, Yang DC et al. PlantTFDB 4.0: toward a central hub for transcription factors and regulatory interactions in plants, Nucleic Acids Research 2017;45:D1040-d1045.
25. Pedruzzi I, Rivoire C, Auchincloss AH et al. HAMAP in 2015: updates to the protein family classification and annotation system, Nucleic Acids Research 2015;43:D1064-1070.
26. Gaudet P, Michel PA, Zahn-Zabal M et al. The neXtProt knowledgebase on human proteins: 2017 update, Nucleic Acids Research 2017;45:D177-d182.
27. Li S, Shui K, Zhang Y. CGDB: a database of circadian genes in eukaryotes, Nucleic Acids Research 2017;45:D397-d403.
28. Franco-Serrano L, Hernández S, Calvo A et al. MultitaskProtDB-II: an update of a database of multitasking/moonlighting proteins, Nucleic Acids Research 2018;46:D645-d648.
29. Ribeiro DM, Briere G, Bely B et al. MoonDB 2.0: an updated database of extreme multifunctional and moonlighting proteins, Nucleic Acids Research 2019;47:D398-d402.
30. Giurgiu M, Reinhard J, Brauner B et al. CORUM: the comprehensive resource of mammalian protein complexes-2019, Nucleic Acids Research 2019;47:D559-d563.
31. Zhang X, Lan Y, Xu J et al. CellMarker: a manually curated resource of cell markers in human and mouse, Nucleic Acids Research 2019;47:D721-d728.
32. Pándy-Szekeres G, Munk C, Tsonkov TM et al. GPCRdb in 2018: adding GPCR structure models and ligands, Nucleic Acids Research 2018;46:D440-d446.
33. Liao JY, Yang B, Zhang YC et al. EuRBPDB: a comprehensive resource for annotation, functional and oncological investigation of eukaryotic RNA binding proteins (RBPs), Nucleic Acids Research 2020;48:D307-d313.
34. Burley SK, Berman HM, Bhikadiya C et al. RCSB Protein Data Bank: biological macromolecular structures enabling research and education in fundamental biology, biomedicine, biotechnology and energy, Nucleic Acids Research 2019;47:D464-d474.
35. Andreeva A, Howorth D, Chothia C et al. SCOP2 prototype: a new approach to protein structure mining, Nucleic Acids Research 2014;42:D310-314.
36. Dosztányi Z, Csizmok V, Tompa P et al. IUPred: web server for the prediction of intrinsically unstructured regions of proteins based on estimated energy content, Bioinformatics 2005;21:3433-3434.
37. Hatos A, Hajdu-Soltész B, Monzon AM et al. DisProt: intrinsic protein disorder annotation in 2020, Nucleic Acids Research 2020;48:D269-d276.
38. Sagendorf JM, Markarian N, Berman HM et al. DNAproDB: an expanded database and web-based tool for structural analysis of DNA-protein complexes, Nucleic Acids Research

2020;48:D277-d287.
39. Kawashima S, Pokarowski P, Pokarowska M et al. AAindex: amino acid index database, progress report 2008, Nucleic Acids Research 2008;36:D202-205.
40. Wilkins MR, Gasteiger E, Bairoch A et al. Protein identification and analysis tools in the ExPASy server, Methods Mol Biol 1999;112:531-552.
41. El-Gebali S, Mistry J, Bateman A et al. The Pfam protein families database in 2019, Nucleic Acids Research 2019;47:D427-d432.
42. Sigrist CJ, de Castro E, Cerutti L et al. New and continuing developments at PROSITE, Nucleic Acids Research 2013;41:D344-347.
43. Mitchell AL, Attwood TK, Babbitt PC et al. InterPro in 2019: improving coverage, classification and access to protein sequence annotations, Nucleic Acids Research 2019;47:D351-d360.
44. Nikolskaya AN, Arighi CN, Huang H et al. PIRSF family classification system for protein functional and evolutionary analysis, Evol Bioinform Online 2007;2:197-209.
45. Attwood TK, Coletta A, Muirhead G et al. The PRINTS database: a fine-grained protein sequence annotation and analysis resource--its status in 2012, Database (Oxford) 2012;2012:bas019.
46. Lin S, Wang C, Zhou J et al. EPSD: a well-annotated data resource of protein phosphorylation sites in eukaryotes, Brief Bioinform 2021;22:298-307.
47. Xu H, Zhou J, Lin S et al. PLMD: An updated data resource of protein lysine modifications, J Genet Genomics 2017;44:243-250.
48. Huang KY, Lee TY, Kao HJ et al. dbPTM in 2019: exploring disease association and cross-talk of post-translational modifications, Nucleic Acids Research 2019;47:D298-d308.
49. Hornbeck PV, Zhang B, Murray B et al. PhosphoSitePlus, 2014: mutations, PTMs and recalibrations, Nucleic Acids Research 2015;43:D512-520.
50. Huang H, Arighi CN, Ross KE et al. iPTMnet: an integrated resource for protein post-translational modification network discovery, Nucleic Acids Research 2018;46:D542-d550.
51. Goel R, Harsha HC, Pandey A et al. Human Protein Reference Database and Human Proteinpedia as resources for phosphoproteome analysis, Mol Biosyst 2012;8:453-463.
52. Landrum MJ, Lee JM, Benson M et al. ClinVar: improving access to variant interpretations and supporting evidence, Nucleic Acids Research 2018;46:D1062-d1067.
53. Li MJ, Liu Z, Wang P et al. GWASdb v2: an update database for human genetic variants identified by genome-wide association studies, Nucleic Acids Research 2016;44:D869-876.
54. Schaefer C, Meier A, Rost B et al. SNPdbe: constructing an nsSNP functional impacts database, Bioinformatics 2012;28:601-602.
55. Krassowski M, Paczkowska M, Cullion K et al. ActiveDriverDB: human disease mutations and genome variation in post-translational modification sites of proteins, Nucleic Acids Research 2018;46:D901-d910.
56. Dingerdissen HM, Torcivia-Rodriguez J, Hu Y et al. BioMuta and BioXpress: mutation and expression knowledgebases for cancer biomarker discovery, Nucleic Acids Research 2018;46:D1128-d1136.
57. Simonetti FL, Tornador C, Nabau-Moretó N et al. Kin-Driver: a database of driver mutations in protein kinases, Database (Oxford) 2014;2014:bau104.
58. Amberger JS, Bocchini CA, Scott AF et al. OMIM.org: leveraging knowledge across phenotype-gene relationships, Nucleic Acids Research 2019;47:D1038-d1043.

59. Xu H, Wang Y, Lin S et al. PTMD: A Database of Human Disease-associated Post-translational Modifications, Genomics Proteomics Bioinformatics 2018;16:244-251.
60. Yue M, Zhou D, Zhi H et al. MSDD: a manually curated database of experimentally supported associations among miRNAs, SNPs and human diseases, Nucleic Acids Research 2018;46:D181-d185.
61. Piñero J, Bravo À, Queralt-Rosinach N et al. DisGeNET: a comprehensive platform integrating information on human disease-associated genes and variants, Nucleic Acids Research 2017;45:D833-d839.
62. Zhang G, Shi J, Zhu S et al. DiseaseEnhancer: a resource of human disease-associated enhancer catalog, Nucleic Acids Research 2018;46:D78-d84.
63. Kotlyar M, Pastrello C, Malik Z et al. IID 2018 update: context-specific physical protein-protein interactions in human, model organisms and domesticated species, Nucleic Acids Research 2019;47:D581-d589.
64. Oughtred R, Stark C, Breitkreutz BJ et al. The BioGRID interaction database: 2019 update, Nucleic Acids Research 2019;47:D529-d541.
65. Razick S, Magklaras G, Donaldson IM. iRefIndex: a consolidated protein interaction database with provenance, BMC Bioinformatics 2008;9:405.
66. Cowley MJ, Pinese M, Kassahn KS et al. PINA v2.0: mining interactome modules, Nucleic Acids Research 2012;40:D862-865.
67. Das J, Yu H. HINT: High-quality protein interactomes and their applications in understanding human disease, BMC Syst Biol 2012;6:92.
68. Calderone A, Castagnoli L, Cesareni G. mentha: a resource for browsing integrated protein-interaction networks, Nature Methods 2013;10:690-691.
69. Li T, Wernersson R, Hansen RB. A scored human protein-protein interaction network to catalyze genomic interpretation, Nature Methods 2017;14:61-64.
70. Szklarczyk D, Gable AL, Lyon D et al. STRING v11: protein-protein association networks with increased coverage, supporting functional discovery in genome-wide experimental datasets, Nucleic Acids Research 2019;47:D607-d613.
71. Higueruelo AP, Jubb H, Blundell TL. TIMBAL v2: update of a database holding small molecules modulating protein-protein interactions, Database (Oxford) 2013;2013:bat039.
72. Li YH, Yu CY, Li XX et al. Therapeutic target database update 2018: enriched resource for facilitating bench-to-clinic research of targeted therapeutics, Nucleic Acids Research 2018;46:D1121-d1127.
73. Wishart DS, Feunang YD, Guo AC et al. DrugBank 5.0: a major update to the DrugBank database for 2018, Nucleic Acids Research 2018;46:D1074-d1082.
74. Harding SD, Sharman JL, Faccenda E et al. The IUPHAR/BPS Guide to PHARMACOLOGY in 2018: updates and expansion to encompass the new guide to IMMUNOPHARMACOLOGY, Nucleic Acids Research 2018;46:D1091-d1106.
75. Zhang JX, Huang WJ, Zeng JH et al. DITOP: drug-induced toxicity related protein database, Bioinformatics 2007;23:1710-1712.
76. Verbruggen B, Gunnarsson L, Kristiansson E et al. ECOdrug: a database connecting drugs and conservation of their targets across species, Nucleic Acids Research 2018;46:D930-d936.
77. Cotto KC, Wagner AH, Feng YY et al. DGIdb 3.0: a redesign and expansion of the drug-gene interaction database, Nucleic Acids Research 2018;46:D1068-d1073.

78. Davis AP, Grondin CJ, Johnson RJ et al. The Comparative Toxicogenomics Database: update 2019, Nucleic Acids Research 2019;47:D948-d954.
79. Ursu O, Holmes J, Bologa CG et al. DrugCentral 2018: an update, Nucleic Acids Research 2019;47:D963-d970.
80. Sonnhammer EL, Östlund G. InParanoid 8: orthology analysis between 273 proteomes, mostly eukaryotic, Nucleic Acids Research 2015;43:D234-239.
81. Altenhoff AM, Glover NM, Train CM et al. The OMA orthology database in 2018: retrieving evolutionary relationships among all domains of life through richer web and programmatic interfaces, Nucleic Acids Research 2018;46:D477-d485.
82. Kriventseva EV, Kuznetsov D, Tegenfeldt F et al. OrthoDB v10: sampling the diversity of animal, plant, fungal, protist, bacterial and viral genomes for evolutionary and functional annotations of orthologs, Nucleic Acids Research 2019;47:D807-d811.
83. Kanehisa M, Sato Y, Furumichi M et al. New approach for understanding genome variations in KEGG, Nucleic Acids Research 2019;47:D590-d595.
84. Fazekas D, Koltai M, Türei D et al. SignaLink 2 - a signaling pathway resource with multi-layered regulatory networks, BMC Syst Biol 2013;7:7.
85. Wishart DS, Li C, Marcu A et al. PathBank: a comprehensive pathway database for model organisms, Nucleic Acids Research 2020;48:D470-d478.
86. Fabregat A, Jupe S, Matthews L et al. The Reactome Pathway Knowledgebase, Nucleic Acids Research 2018;46:D649-d655.
87. Han H, Cho JW, Lee S et al. TRRUST v2: an expanded reference database of human and mouse transcriptional regulatory interactions, Nucleic Acids Research 2018;46:D380-d386.
88. Wang Z, Zhang Q, Zhang W et al. HEDD: Human Enhancer Disease Database, Nucleic Acids Research 2018;46:D113-d120.
89. Murali T, Pacifico S, Yu J et al. DroID 2011: a comprehensive, integrated resource for protein, transcription factor, RNA and gene interactions for Drosophila, Nucleic Acids Research 2011;39:D736-743.
90. Yang TH, Wang CC, Wang YC et al. YTRP: a repository for yeast transcriptional regulatory pathways, Database (Oxford) 2014;2014:bau014.
91. Liu ZP, Wu C, Miao H et al. RegNetwork: an integrated database of transcriptional and post-transcriptional regulatory networks in human and mouse, Database (Oxford) 2015;2015.
92. Athar A, Füllgrabe A, George N et al. ArrayExpress update - from bulk to single-cell expression data, Nucleic Acids Research 2019;47:D711-d715.
93. Smith CM, Hayamizu TF, Finger JH et al. The mouse Gene Expression Database (GXD): 2019 update, Nucleic Acids Research 2019;47:D774-d779.
94. Kim P, Park A, Han G et al. TissGDB: tissue-specific gene database in cancer, Nucleic Acids Research 2018;46:D1031-d1038.
95. Zhang Z, Townsend JP. The filamentous fungal gene expression database (FFGED), Fungal Genetics and Biology 2010;47:199-204.
96. Palasca O, Santos A, Stolte C et al. TISSUES 2.0: an integrative web resource on mammalian tissue expression, Database (Oxford) 2018;2018.
97. Uhlen M, Zhang C. A pathology atlas of the human cancer transcriptome, Science 2017;357.
98. Kim MS, Pinto SM, Getnet D et al. A draft map of the human proteome, Nature 2014;509:575-581.

99. Glažar P, Papavasileiou P, Rajewsky N. circBase: a database for circular RNAs, RNA 2014;20:1666-1670.
100. Huang W, Ling Y, Zhang S et al. TransCirc: an interactive database for translatable circular RNAs based on multi-omics evidence, Nucleic Acids Research 2021;49:D236-d242.
101. Agarwal V, Bell GW, Nam JW et al. Predicting effective microRNA target sites in mammalian mRNAs, Elife 2015;4.
102. Sticht C, De La Torre C, Parveen A et al. miRWalk: An online resource for prediction of microRNA binding sites, PLoS One 2018;13:e0206239.
103. Jeggari A, Marks DS, Larsson E. miRcode: a map of putative microRNA target sites in the long non-coding transcriptome, Bioinformatics 2012;28:2062-2063.
104. Lin Y, Liu T, Cui T et al. RNAInter in 2020: RNA interactome repository with increased coverage and annotation, Nucleic Acids Research 2020;48:D189-d197.
105. Chen C, Zhou D, Gu Y et al. SEA version 3.0: a comprehensive extension and update of the Super-Enhancer archive, Nucleic Acids Research 2020;48:D198-d203.
106. Hsu PW, Huang HD, Hsu SD et al. miRNAMap: genomic maps of microRNA genes and their target genes in mammalian genomes, Nucleic Acids Research 2006;34:D135-139.
107. Xiao F, Zuo Z, Cai G et al. miRecords: an integrated resource for microRNA-target interactions, Nucleic Acids Research 2009;37:D105-110.
108. Chou CH, Shrestha S, Yang CD et al. miRTarBase update 2018: a resource for experimentally validated microRNA-target interactions, Nucleic Acids Research 2018;46:D296-d302.
109. Bernhofer M, Goldberg T, Wolf S et al. NLSdb-major update for database of nuclear localization signals and nuclear export signals, Nucleic Acids Research 2018;46:D503-d508.
110. Binder JX, Pletscher-Frankild S, Tsafou K et al. COMPARTMENTS: unification and visualization of protein subcellular localization evidence, Database (Oxford) 2014;2014:bau012.
111. Lomize AL, Hage JM, Pogozheva ID. Membranome 2.0: database for proteome-wide profiling of bitopic proteins and their dimers, Bioinformatics 2018;34:1061-1062.
112. Mendik P, Dobronyi L, Hári F et al. Translocatome: a novel resource for the analysis of protein translocation between cellular organelles, Nucleic Acids Research 2019;47:D495-d505.

Supplementary Figures

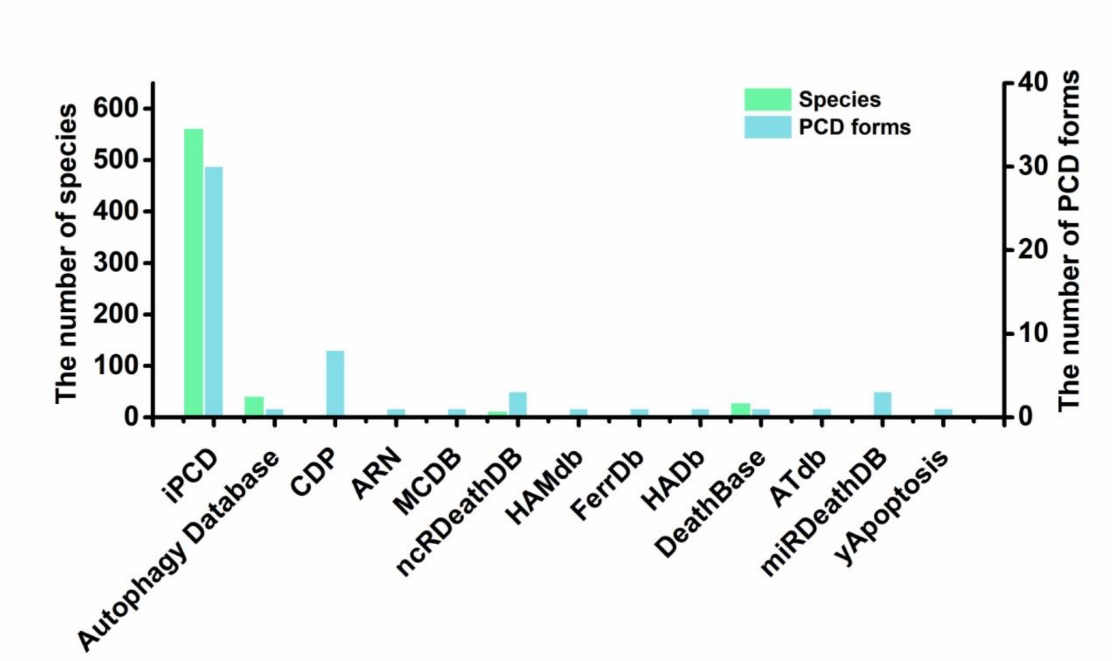

Figure S1. The numbers of PCD types and species in iPCD and 12 databases. The detailed information of 12 databases were presented in the Supplementary Table S1.

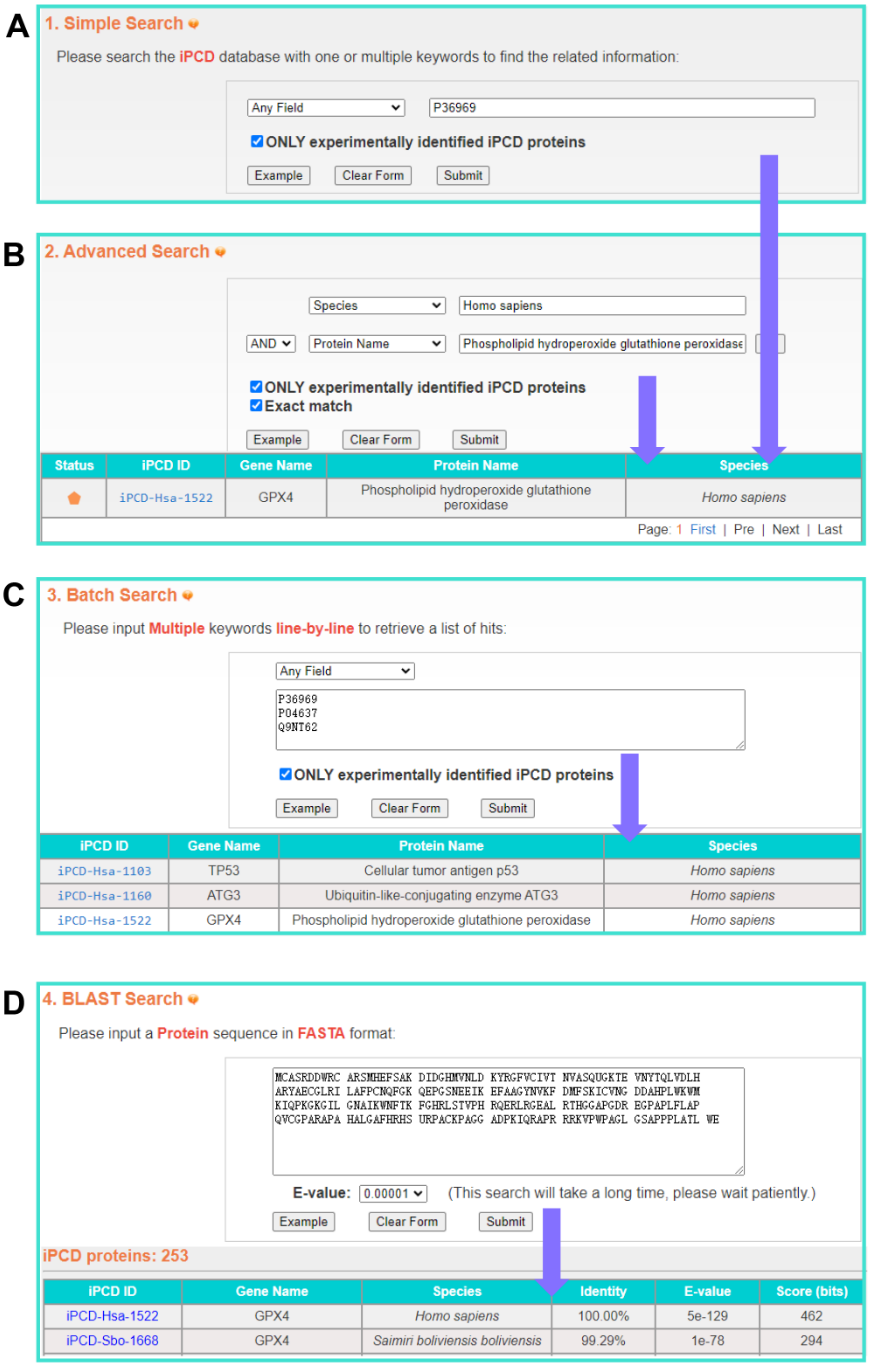

Figure S2. The four search options of iPCD, including (A) 'Simple Search' can show specific information of a protein by inputting a keyword, (B) 'Advanced Search' allows to find more information with multiple terms, (C) 'Batch Search' with a list keywords in a line-by-line format, and (D) 'BLAST Search' using a protein sequence in FASTA format.

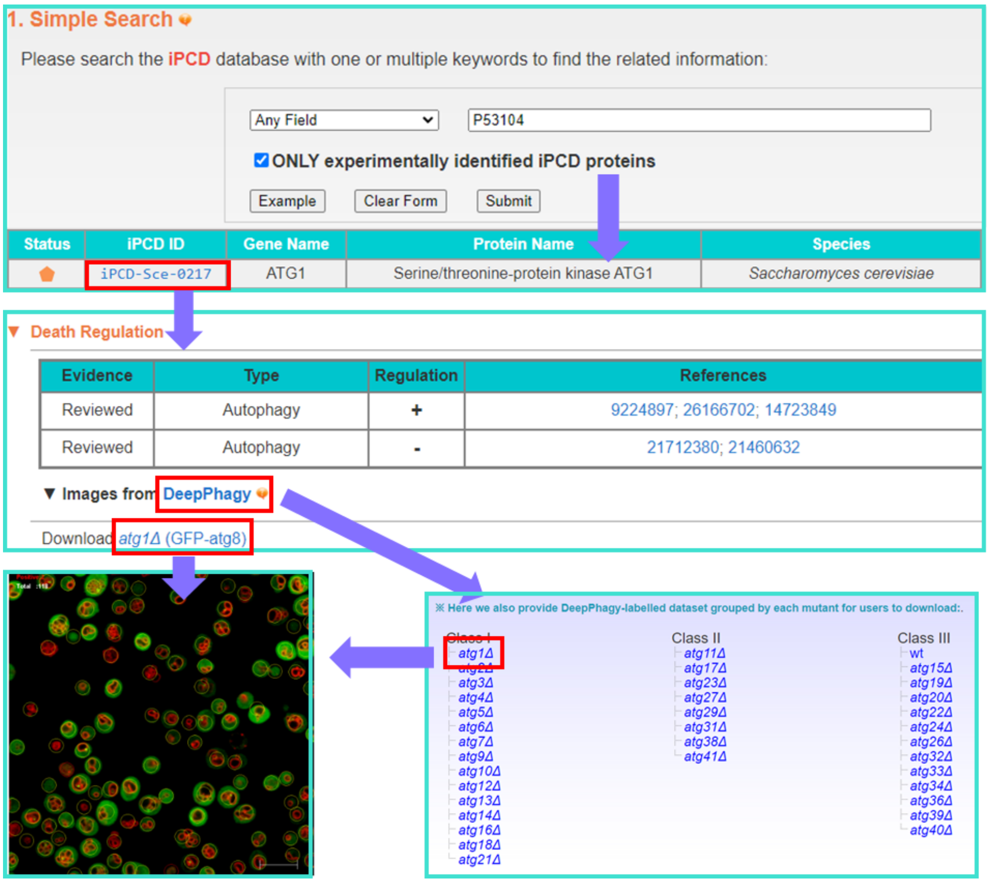

Figure S3. The fluorescence images of *S. cerevisiae* for 35 ATGs mutants can be download by clicking on the 'DeepPhagy' button or clicking on the corresponding atgs section.

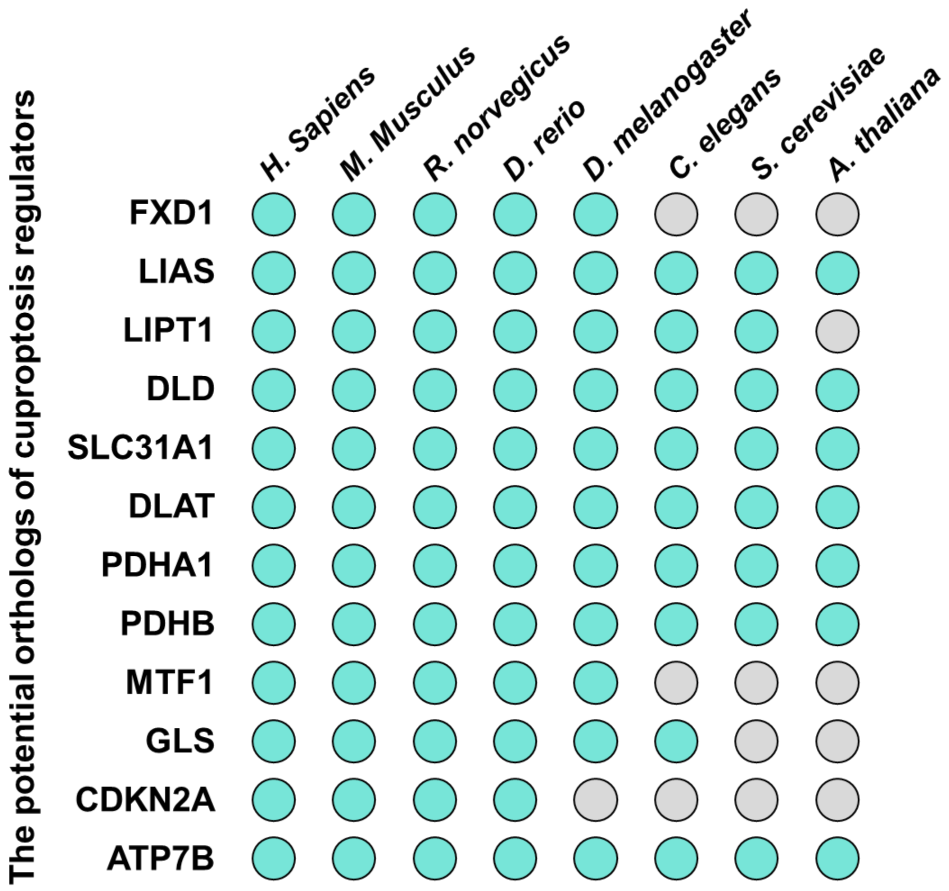

Figure S4. The potential orthologs of 12 cuproptosis regulators were computationally identified in 8 model organisms. The existent of cuproptosis potential regulators were marked with a green ball.

Supplementary Tables

Table S1. A brief summary of 12 PCD resources. *a*. The criteria or methods used for data collection, including literature curation, taking from proteomic profiling analyses (proteomic profiling), integration from other public databases (database integration), and computational prediction.

Table S2. The distribution of numbers of experimentally identified PCD regulators, which were newly collected into iPCD and not covered by other public PCD resources.

Table S3. The detailed summary of 30 PCD forms.

Table S4. The annotation of iPCD from 102 public resources covered 16 aspects.

Table S5. The distribution of regulatory proteins and corresponding PCD forms in 562 eukaryotes.

Table S6. The data statistics of experimentally identified proteins for 30 PCD forms in 8 model organisms.

Table S7. The data statistics of human regulators involved different forms of PCD.
